# GraS signaling in *Staphylococcus aureus* is regulated by a single D35 residue in the extracellular loop

**DOI:** 10.1128/spectrum.01982-23

**Published:** 2023-09-20

**Authors:** Junho Cho, Adhar C. Manna, Helah S. Snelling, Ambrose L. Cheung

**Affiliations:** 1 Department of Microbiology and Immunology, Geisel School of Medicine, Dartmouth College, Hanover, New Hampshire, USA; CCG-UNAM, Cuernavaca, Mexico

**Keywords:** MRSA, two-component regulatory systems, antimicrobial peptides, GraRS TCS

## Abstract

Bacterial two-component systems are crucial features of bacterial pathogens such as methicillin-resistant *Staphylococcus aureus* to overcome environmental and antimicrobial stresses by activating regulons to interfere with the bactericidal mechanisms. GraRS is a unique subset of two-component systems belonging to the intramembrane-sensing histidine kinase family (IM-HK) and is responsible for resistance to cationic host defense peptides. However, the precise manner by which the short 9-residue extracellular loop of the membrane sensor GraS detects the antimicrobial peptides and transduces the signal is not comprehensively understood. Here, we show that a single point mutation (D35A) in the extracellular loop of GraS blocked activation of GraRS, but this effect was also abrogated with *graS* mutations in the N-terminal transmembrane segments without any accompanying effect on GraS protein expression. Additionally, mutations in H120 and T172 in the dimerization/histidine phosphotransfer (DHp) domain of GraS increased activation without any accompanying enhancement in dimerization, likely due to disruption of the H120-T172 interaction that restricts rotational movements of the DHp helices since swapping H120 and T172 did not alter GraS activation. Notably, the enhancing effects of H120 and T172 mutations were abolished with a D35 mutation, highlighting the pivotal role of D35 in the 9-residue extracellular loop of GraS in GraR phosphorylation. In summary, our study delivers the significance of the D35 in the extracellular loop of GraS and ensuing changes in the N-terminal transmembrane helices as a model to illustrate signaling in the IM-HK subset of two-component regulatory systems.

**IMPORTANCE:**

Methicillin-resistant *Staphylococcus aureus* (MRSA) is a human pathogen capable of infecting skin, blood, internal organs, and artificial medical devices. Generally, personal hygiene and a robust immune system can limit the spread of this pathogen; however, MRSA possesses an assortment of phenotypic tools to survive the hostile host environment including host defense peptides. More specifically, *S. aureus* utilizes two-component systems to sense noxious environmental cues to respond to harmful environmental elements. Our study focused on a two-component system called GraRS that *S. aureus* deploys against host defense peptides. We showed that one single residue in the extracellular loop of GraS and the adjacent membrane segment controlled the activation of GraRS, indicating the importance of a well-tuned-charged residue in the extracellular loop of GraS for sensing activity.

## INTRODUCTION

A crucial element in host defense against *Staphylococcus aureus* is innate immunity, which entails anatomic defense, cytokines, polymorphonuclear neutrophils (PMNs), and proteins such as complement and “host defense peptides” (HDPs) (1, 2). HDPs, aka defensins, stand for a class of potent cationic antimicrobial peptides that kill bacteria by inserting into bacterial membranes followed by multimerization and eventual pore formation. The response of *S. aureus* to HDPs is propelled by two-component regulatory systems (TCS) comprising the GraS histidine kinase (HK) and its cognate response regulator (GraR) (3). The model predicts that GraS is activated by its cognate environmental stimulus (e.g., HDPs), leading to autophosphorylation of the HK sensor GraS and ensuing phosphor-transfer to GraR and finally expression of specific bacterial defense proteins (e.g., MprF in response to HDPs) to increase the surface positive charge to dissuade interaction with HDPs (4).

Among the ~2,000 HKs identified from 350 species of Gram-positive bacteria (majority), Proteobacteria, and Actinobacteria, the sensor kinase GraS belongs to a unique subset (~150 genomes) called intramembrane-sensing HK (IM-HK), wherein each HK comprises two transmembrane (TM) helices framing a very short extracellular loop (EL) (often <10 A.A.) for sensing (4). While it has been proposed that IM-HKs sense via neighboring membrane perturbation (e.g., from HDP binding), definitive proof of this mechanism is lacking. In addition, a large group of IM-HKs have been found to harbor genetic and topological links to adjacent ATP-binding cassette (ABC) transporter genes, *vraFG* (e.g., *graRS-vraFG*). We recently showed that the 200-residue EL of the membrane permease VraG likely interacts with GraS to regulate GraS-mediated sensing (5).

The cytosolic portion of the GraS sensor protein encompasses regions that include a domain found in histidine kinases, adenylyl cyclases, methyl-accepting proteins, and phosphatases (HAMP); in addition, a dimerization/histidine phosphotransfer (DHp) domain and an ATPase domain are found on its C-terminus, while hooked transmembrane helices containing a short sensing EL are located on its N-terminus. With the exception of the very small EL, this topology is found in many prokaryotic HKs within the IM-HK family. Upon activation, GraS triggers a phosphor-relay onto GraR (response regulator, RR), which then prompts the expression of *mprF* and *dltABCD*, two loci involved in the increase of relative surface positive charge by adding lysine and alanine to the membrane phosphatidylglycerol and teichoic acids, respectively, to repel cationic HDPs (3, 6 – 8). An important question to ask is how GraS can sense HDPs with its short 9-residue EL and transfer its signal to GraR.

Here, we present data to show that one aspartate residue (D35) in the EL of GraS governs activation, likely by affecting the conformation of the TM helix. As predicted, mutation of the aspartate residue to lysine or alanine significantly decreased *mprF* expression, reduced surface positive charge, and diminished survival upon 2 h LL37 exposure vs the non-mutated control. More importantly, these genotypic and phenotypic changes were also observed with mutations of transmembrane helices in GraS without any noticeable defect in protein expression. Another fascinating finding was that the ability of GraS to dimerize did not correlate with activation of GraS because the D35K mutation in GraS disrupted GraS signaling but did not alter dimer formation. We also found that H120A and T172A mutations in the DHp domain enhanced GraS signaling without affecting dimerization. These results counter the conventional view that “dimerization of HK with specific stimuli leads to activation” (9, 10). Finally, we found that the D35 mutation utterly disrupted the activation of GraS caused by H120A and T172A mutations. In the phosphorylation assay of the response protein GraR, the *graS* D35K mutation, along with H120A, T172A, or double H120A/T172A mutation, disclosed a lower level of GraR phosphorylation vs H120A and/or T172A mutant, correlating with reduced *mprF* expression, increased cytochrome c binding, and enhanced LL-37 mediated killing. Collectively, our results indicate that D35 in the EL of GraS, along with well-coordinated transmembrane helices, is essential for initiating and controlling the GraRS TCS system.

## RESULTS

### A single-point mutation in the EL of GraS blocks activation of GraS

**Fig 1:**
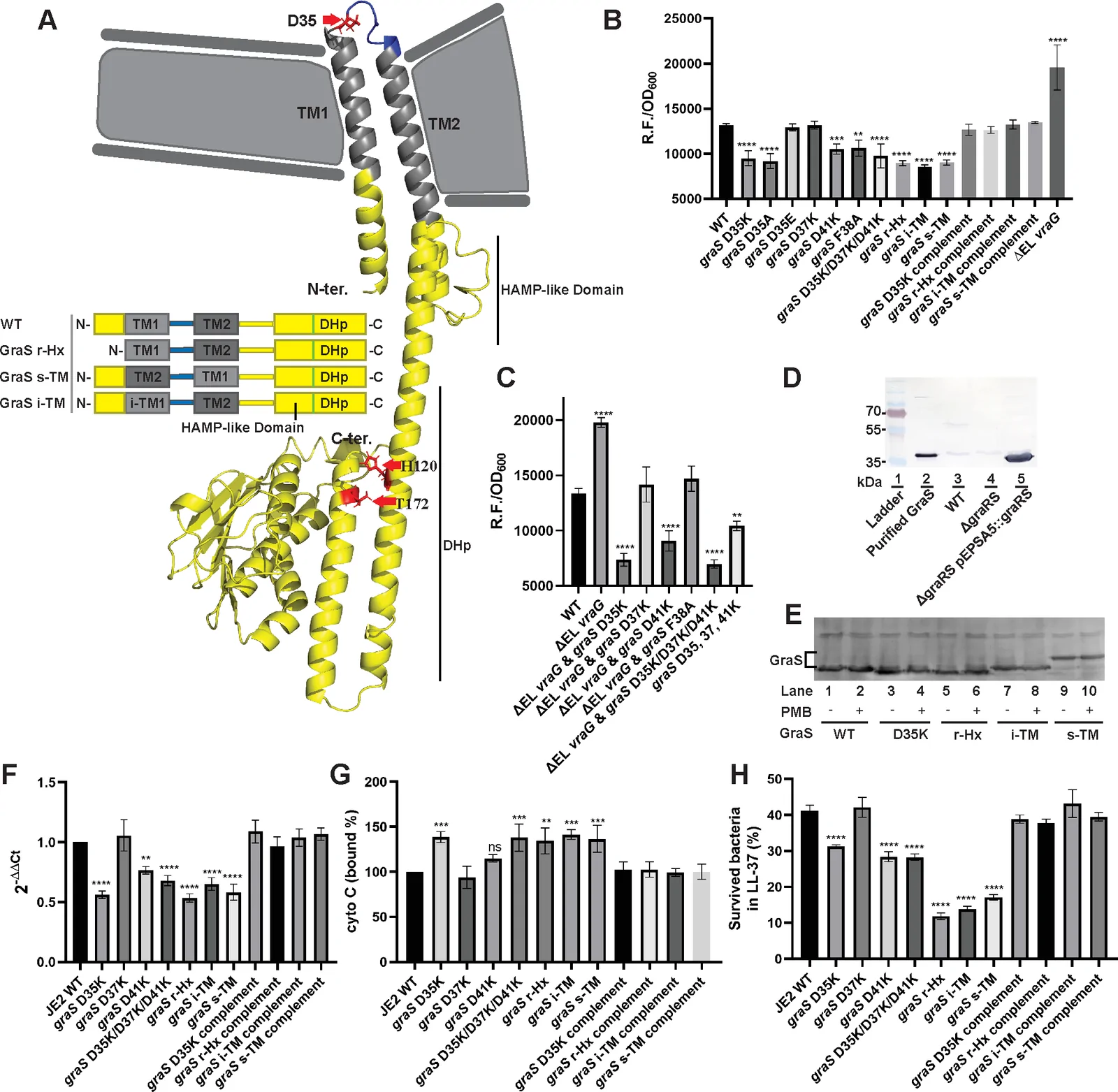
Mutations in GraS EL and TMs block GraS signaling. (**A**) Predicted GraS structure. The GraS structure was predicted using the RoseTTAFold modeling and visualized using PyMol (version 2.0.7) (12). GraS D35, H120, and T172 residues were depicted with a red stick. The transmembrane helices and cytosolic domains were colored gray and yellow, respectively. A simplified cartoon for the *graS* mutants was illustrated on the left. (**B**) and (**C**) GPFuvr expression driven by the *mprF* promoter. The mutants with pALC1484::*mprF* promoter grown to the mid-log phase were monitored for fluorescent intensities and OD_600_ (mid-log phase). The *vraG* ΔEL mutant represents deletion of the extracellular loop in *vraG* gene (5). D37K and parental strain were used as the unperturbed controls. (**D**) Western blotting with GraS antibody that we have used previously (11). Lane 1: ladder, lane 2: purified GraS, lane 3: the wild-type strain, lane 4: Δ*graRS,* and lane 5: Δ*graRS* with pEPSA5::*graRS.* (**E**) Western blotting with GraS antibody for D35K, TM, and N-terminal helical deletion mutant. Due to the expression issue with *graS* mutants, we used Δ*graXRS* with pEPSA5::*graXRS*, which overexpresses GraX along with GraR and GraS. Lanes 1 and 2: Δ*graXRS* with pEPSA5::*graXRS*, lanes 3 and 4: Δ*graXRS* with pEPSA5::*graXRS graS* D35K*,* lanes 5 and 6: Δ*graXRS* with pEPSA5::*graXRS graS* r-Hx*,* lanes 7 and 8: Δ*graXRS* with pEPSA5::*graXRS graS* i-TM*,* and lanes 9 and 10: Δ*graXRS* with pEPSA5::*graXRS graS* s-Tm. The samples in even lanes were induced with 64 µg/mL PMB for 30 min. (**F**) Quantification of *mprF* transcript through qRT-PCR. We used specific primers (*gyrB* and *mprF*) to reverse-transcribe RNAs, enabling us to obtain consistent values for selectively enriched cDNAs. (**G**) Cytochrome c binding to the cell surface. The number of cells was normalized to a fixed value (OD_650_ ~3) and treated with a constant concentration of cytochrome c (0.25 mg/mL). After a short incubation, we estimated the amount of the bound cytochrome c to the cell surface by subtracting the unbound amount from the initial amount of cytochrome c. The amount of cytochrome c bound to the parent JE2 was set at 100%. (**H**) LL-37 susceptibility assay. Cells grown in BHI were washed with phosphate buffer and treated with freshly prepared LL-37 at 2.5 mg/mL for 2 h at 37°C. The number of surviving cells was counted and compared to the number of each inoculum. The results for the LL-37 susceptibility assay are representative of independent experiments. The values from at least three biological replicates were analyzed using one-way ANOVA vs wild type for other assays (****P < 0.0001, 0.0001≤***P<0.001, 0.001≤**P<0.01, 0.01≤*P<0.05, and ns or not labeled considered non-significant with *P* ≥ 0.05).

GraS encompasses two transmembrane segments with cytosolic tails framing a 9-residue EL (depicted as a blue loop in Fig. 1A), which senses positively charged HDPs and transduces the signal to the response protein GraR via a phosphor-relay. Within the 9-residue EL (DYDFPIDSL) are three negatively charged aspartate residues. Previous studies have shown that these three aspartic acid residues (D35, 37, and 41) play a vital role in GraS signaling, as supported by data showing that a triple D35K/D37K/D41K mutant lost its ability to activate GraS (11).

To investigate the contribution of each of these aspartate residues, individual D35K, D37K, D41K, or F38A *graS* mutants were constructed using allelic exchange with the pMAD vector (13) (Tables S1 and S2 in the supplemental material). Using a reporter plasmid containing the *mprF* promoter fused to *gfpuvr* (pALC1484), we assessed *mprF* expression in these mutants vs the parent JE2 in the absence of CAMPs. For the positive control, we utilized a *vraG* mutant lacking its extracellular loop, which has been shown to over-express *mprF* (5). As shown in Fig. 1B, the wild-type JE2 strain had a basal expression of ~13,000 fluorescence unit per OD_600_ (obtained at log phase corresponding to OD_600_ of ~0.6) (Fig. 1B). The *graS* D35K and D35A mutants had the lowest GFPuvr expression vs the parent, parallel to what was observed in the triple D35K/D37K/D41K mutant. In contrast, the *graS* D37K mutant displayed *mprF* expression level similar to the wild type, while the *graS* D41K mutant displayed *mprF* expression at an intermediate level between the parent and the D35K or D35A mutants. In the presence of polymyxin B (PMB), *mprF* expression was increased in the wild type while Δ*graS* and *graS* D35K mutants were not affected by the PMB stress (Fig. S1 in the supplemental material). We previously reported that mutation at F38 was not as effective as the aspartate mutation for signal transduction (11); in the current study, we also observed a conspicuous reduction in *mprF* expression vs the parent but higher than the D35K or D35A mutant. Interestingly, the D35E mutant (no charge alteration but bulkier residue) exhibited a level of *mprF* expression similar to the wild type (Fig. 1B). Together, these data imply that the negative charge of the residue at position 35 in the EL played a pivotal role in GraS-mediated signal transduction. However, as *mprF* expression was comparable between the D35K (positive charge) and D35A (neutral charge) mutant, it also implied that charge change alone cannot account for the defect (about 25% reduction vs the wild type) in *mprF* expression in the D35K mutant.

As an IM-HK, *graRS* was found adjacent to *vraFG* encoding an ATP-dependent efflux pump (*graRS-vraFG*). In a previous study, we showed that the 200-residue long EL of VraG (encoding a permease) interferes with GraS sensing since a ΔEL *vraG* mutant enhanced GraS-mediated *mprF* expression (5). We wondered if activation of *mprF* in the ΔEL *vraG* mutant requires D35 in the EL for optimal tuning of GraS. Remarkably, introduction of D35K or D35K/D37K/D41K mutation into the ΔEL *vraG* mutant significantly lowered *mprF* expression to the level of the D35K or the D35K/D37K/D41K mutant, while the ΔEL *vraG* mutant alone yielded higher *mprF* expression than the parent (Fig. 1C). Notably, there appeared to be a hierarchy in the EL aspartates in *mprF* expression, with the effect of D35 being the most prominent, whereas D37 has almost no effect (D35 > D41 > D37). Introducing the F38A mutation into the ΔEL *vraG* mutant reduced *mprF* expression to the wild-type level, similar to the *graS* D37 mutant (Fig. 1C). Collectively, these data signify that D35 in the EL of GraS is a crucial residue for *mprF* activation.

The D35 residue within the EL of GraS is predicted to be located at the end of the EL adjacent to the N-terminal transmembrane helix (TM1) (Fig. S2 in the supplemental material), where the residue is likely to develop repulsive tension against the negatively charged lipid head groups on the surface of the outer membrane. As this tension could be attenuated by the GraS D35 mutation, we envisioned that the interaction between two TMs (TM1 and TM2) might be influenced or altered due to the D35 mutation, eventually leading to changes in cytosolic portions of GraS and hence alteration in signal transduction. To discern the effect of the transmembrane segment on D35-mediated signal transduction, we constructed *graS* mutants having an inverted TM1 (i-TM) and a swapped TM between TM1 and TM2 (s-TM) to interrupt the interaction of TMs, and also a truncated N-terminal helix (r-Hx) to discern if the cytosolic N-terminal helix is necessary for GraS activity (Fig. 1A; Fig. S2 in the supplemental material). The newly constructed mutants were transformed with the vector containing the *mprF* promoter fused to *gfpuvr* gene and compared to the GraS D35K mutant, parent, and complemented mutants (Fig. 1B). As expected, all the membrane mutants appeared to possess lower *mprF* expression similar to the GraS D35K mutant, as compared to the parent and the corresponding complemented mutants, thus suggesting that proper positioning and interaction of the TMs and the presence of the N-terminal cytosolic helix were required for optimal GraS signal transduction. To assess if GraS proteins with the transmembrane mutations were stably expressed, we visualized the native and mutated GraS protein using Western blotting. As shown in Fig. 1D, the native GraS protein was ~35 kDa in size. GraS expression remained unaffected even after the treatment of PMB, further confirming the non-autoregulation trait of GraRS TCS (14). GraS proteins associated with TM and D35K mutations as well as the N-terminal deletion (r-Hx) that all yielded lower *mprF* expression were all expressed (Fig. 1E), but the TM mutations appeared to affect the migration rates of the GraS proteins even in SDS-PAGE (Fig. 1D), possibly due to membrane structural alternations (11, 15) (Fig. S5 and S6 in the supplemental material). These data indicate that, in contrast to the D35K point mutant, GraS proteins associated with TM mutations may lead to structural changes that affect protein migration on SDS-PAGE.

To confirm the effect of EL point mutations, the membrane segments, and the N-terminal portion of GraS on *mprF* expression, we performed qRT-PCR analysis to measure the relative amount of *mprF* transcript in assorted *graS* mutants vs the parent against a housekeeping gene (*gyrB*) and complemented mutants followed by a cytochrome c (positively charged protein) binding assay to monitor the surface membrane charge (Fig. 1F and G) in the absence of CAMPs. The qRT-PCR data appeared to mirror the trends seen with GPFuvr expression mediated by the *mprF* promoter, showing a significant drop in the D35K mutant followed by D41K and no drop in the D37K mutants. The N-terminal deletion mutant (*graS* r-Hx) as well as the inverted membrane segment and swapped membrane mutants (i-TM and s-TM mutants) also exhibited a significant drop (about 40% reduction vs the wild type) in *mprF* expression, similar to the D35K mutant and significantly lower than the parent and complemented mutants (Fig. 1F). Reduced *mprF* expression, leading to a reduction in surface positive charge, is predicted to increase binding by cytochrome c. As shown in Fig. 1G, cytochrome c binding was enhanced in the D35, D41, membrane segment and N-terminal deletion mutants vs the wild type and complemented mutants, at levels corresponding to the *mprF* data. As expected, treatment with PMB increased *mprF* expression and decreased cytochrome c binding in the wild type while Δ*graS* and *graS* D35K mutants were not affected (Fig. S1 in the supplemental material). In order to verify the impact of these mutations on cell viability when exposed to human cationic peptides, we performed a 2 h LL-37 killing assay by measuring cell survivability (Fig. 1H). Consistent with other assays, the GraS D35K, D41K, and D35K/D37K/D41K mutants exhibited reduced survival with LL-37. Similarly, those mutants with N-terminal deletion (r-Hx), inverted TM1 (i-TM), and swapped membrane (s-TM) mutants were more sensitive to LL-37 exposure vs the parent and complemented mutants, displaying lowered survivability vs wild type. Overall, the change in *mprF* expression identified by GFP_uvr_ expression and qRT-PCR was consistently shown to correlate negatively with the cytochrome c-binding assay. The lowered *mprF* expression, leading to reduction in positive cell membrane charge in the *graS* mutants, was most likely to affect the cell viability against LL-37.

### Two residues in DHp of GraS control the activation of GraS

**Fig 2:**
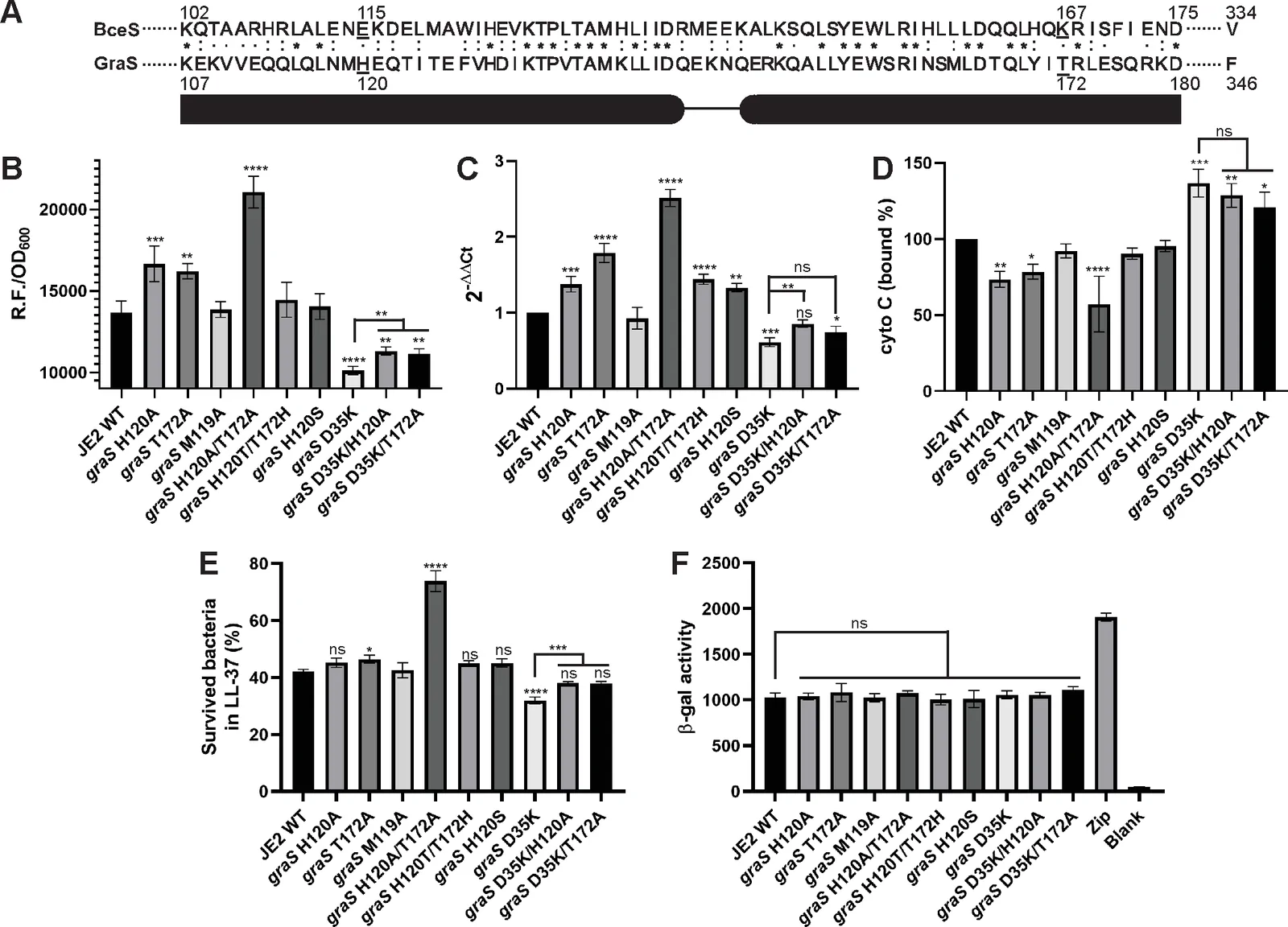
Mutations in GraS DHp domain induce GraS signaling. (**A**) Clustal Omega multiple sequence alignment (17) with GraS and BceS. An asterisk (*) indicates a fully conserved residue with full identity. A colon (:) represents homologous residue with strong similarity with >0.5, and a period (.) implies weak similarity with ≤0.5 in the Gonnet PAM 250. (**B**) and (**C**) Analysis of *mprF* expression through GFP_uvr_ vector and qRT-PCR. H120 and T172 in GraS are homologous residues to E115 and K167 in BceS, respectively, identified from the sequence alignment. M119, located next to H120, was chosen as a neutral control. (**D**) and (**E**) Cytochrome c binding and LL-37 survivability assays. (**F**) BACTH assay to assess dimerization via protein-protein interaction. To analyze the dimerization of GraS, we used *E. coli* strain DHT1 with pKT::*graS* and pUT18::*graS* plasmid constructs (18). The strain was grown overnight and permeabilized to release β-galactosidase. The β-galactosidase activity was measured by the amount of chromophore o-nitrophenol digested from ONPG. For the assays, we analyzed the values obtained from at least three biological replicates using one-way ANOVA, comparing them vs the wild type. In specific cases, we also performed comparisons against the graS D35K mutant when necessary (****P < 0.0001, 0.0001≤***P<0.001, 0.001≤**P<0.01, 0.01≤*P<0.05, and ns or not labeled considered non-significant with *P* ≥ 0.05).

It has been reported that increased helical rotations of the DHp in the HK BceS in *Bacillus subtilis* induce its activation, as supported by a molecular analysis and computational modeling of selected point mutants (16). Since GraRS in *S. aureus* is a homolog of BceRS in *B. subtilis*, we surmised that GraS might possess similar patterns of activation of the DHp domain comparable to BceS. Through sequence alignment analysis with GraS and BceS, we identified and mutated two putative residues (H120 and T172) that may play a role in helical rotations of GraS (Fig. 2A). Using the reporter plasmid that estimated *mprF* expression with the GFP_uvr_ reporter, the *graS* strain with the double H120A/T172A mutation exhibited the highest level of *mprF* expression, while the single H120A and T172A mutants also possessed higher *mprF* level than the wild type but lower than the double H120A/T172A mutant. (Fig. 2B). As a negative control, we deployed the M119A mutant, where the mutated residue lies adjacent to H120, but found no difference in *mprF* expression between the *graS* M119A mutant and the wild type. These trends were upheld on qRT-PCR analysis for *mprF* transcript and cytochrome c-binding assay (Fig. 2C and D). However, survival of the single mutants, H120A and T172A, in LL-37 did not reveal significant differences from the wild type, presumably due to lesser sensitivity of this assay (Fig. 2E) since the double H120A/T172A mutant, with higher level of *mprF* expression vs the single mutant, could survive nearly twice as much as the wild type.

RoseTTAFold modeling suggests that H120 and T172 residues are closely located, hinting at an interaction with each other (Fig. 1A). We surmised that mutations at H120A and/or T172A would disrupt the interaction yet maintaining the molecular architecture (predicted by RoseTAAFold) and allowing free helical rotation and hence activation/auto-phosphorylation of H129 in GraS. If this scenario is correct, we predicted that swapping H120 and T172 (H120T and T172H) might retain their interaction and reduce GraS activation. Hence, we constructed the H120T/T172H mutant and also H120S mutant, which may form hydrogen bond with the threonine residue at position 172 (19). Fluorescence assay of the *mprF* promoter driving GFP_uvr_ expression disclosed comparable levels of fluorescence in H120T/T172H and H120S mutants vs the wild type, in contrast to the H120A and T172A mutants with higher *mprF* expression. However, the qRT-PCR assay, with a higher sensitivity than the promoter fusion assay, revealed that the *graS* H120T/T172H and H120S mutants had higher amounts of *mprF* transcript than the wild type (Fig. 2C) but lower than the H120A/T172A double mutant (Fig. 2A, D and E), along with the cytochrome c binding and LL-37 killing assays that are concordant with the *mprF* expression data, as compared to the H120A/T172A mutant that exhibited higher *mprF* expression than the parent.

### The D35 residue is a major driver of GraS activation without affecting dimerization

Since H120 and T172 residues appeared to regulate GraS activation, we queried if the effect of H120A and T172A mutations on GraS activation could be altered by the D35 mutation. As seen in Fig. 2B and C, the double *graS* D35K/H120A and D35K/T172A mutant displayed reduced *mprF* expression vs the parent and single H120A and T172A mutants, but slightly higher than the D35K mutant. Cytochrome c-binding assay also revealed higher binding in the D35K, D35K/H120A, and D35K/T172A mutant vs the parent and much higher than the H120A/T172A double mutant (Fig. 2D). Concordant with the lesser sensitivity of the LL-37 survivability assay, only the H120A/T172A mutant showed higher survival and the D35K mutant with lower survival than the parent (Fig. 2E). In addition, the triple D35K/H120A/T172A mutant also did not reveal a notable increase in *mprF* expression vs the D35K mutant (Fig. S3 in the supplemental material). These results led to the conclusion that the D35K mutation imposed overwhelming deactivation effects on GraS signaling, regardless of the DHp-activation status by the H120A and/or T172A mutations.

The classic dogma for activation of HK is that a dimer is generally formed, followed by autophosphorylation to initiate the phosphor-relay (20). Although the EL of GraS is located further from the DHp domain, we wanted to evaluate if the D35K mutation, which has been found to curtail GraS activity significantly, would impair GraS dimerization. Using the bacterial adenylate cyclase two-hybrid assay (BACTH) with *E. coli* DHT1 strain, it was found that the D35K mutation did not affect dimerization, at a level similar to the wild type as indicated by the β-galactosidase activity (Fig. 2F; Fig. S4 in the supplemental material). The H120A and T172A mutants also showed no increase in dimerization in comparison to the wild type. These results indicate a lack of correlation between dimerization and (i) the signaling interruption (D35K mutation) or (ii) the rotational movements of the DHp domains (H120A and T172A mutations) at least in IM-HK, a subset of TCS with a small EL in HK sensor.

### The genetic requirement of *graS* in phosphorylation of GraR

We have elucidated above several novel requirements of GraS for activating downstream effectors such as *mprF*. As *mprF* is activated by phosphorylated GraR, we wonder if the phenotypes of the *graS* mutants involving D35, H120, and T172 correlate with GraR phosphorylation. In the course of this study, we have encountered unexpected technical difficulties in purifying GraS. First, the yield for GraS expression and purification has been low. Second, as an IM-HK, GraS is a membrane protein with a short EL and a cytoplasmic tail, making it challenging to purify. After multiple attempts, we found that co-expression with GraX, which was encoded by the operon *graXRS*, was needed to express and purify GraR and mutated GraS proteins. We also appended a C-terminal HA tag to GraR to facilitate detection. Accordingly, we constructed the strains JE2 Δ*graXRS*/pEPASA5::g*raXRS* with various *graS* mutations (Table S1 in the supplemental material). To monitor the phosphorylated GraR, a Phos-tag SDS-PAGE detection system was adopted, in which non-phosphorylated and phosphorylated proteins were allowed to migrate at different rates.

**Fig 3:**
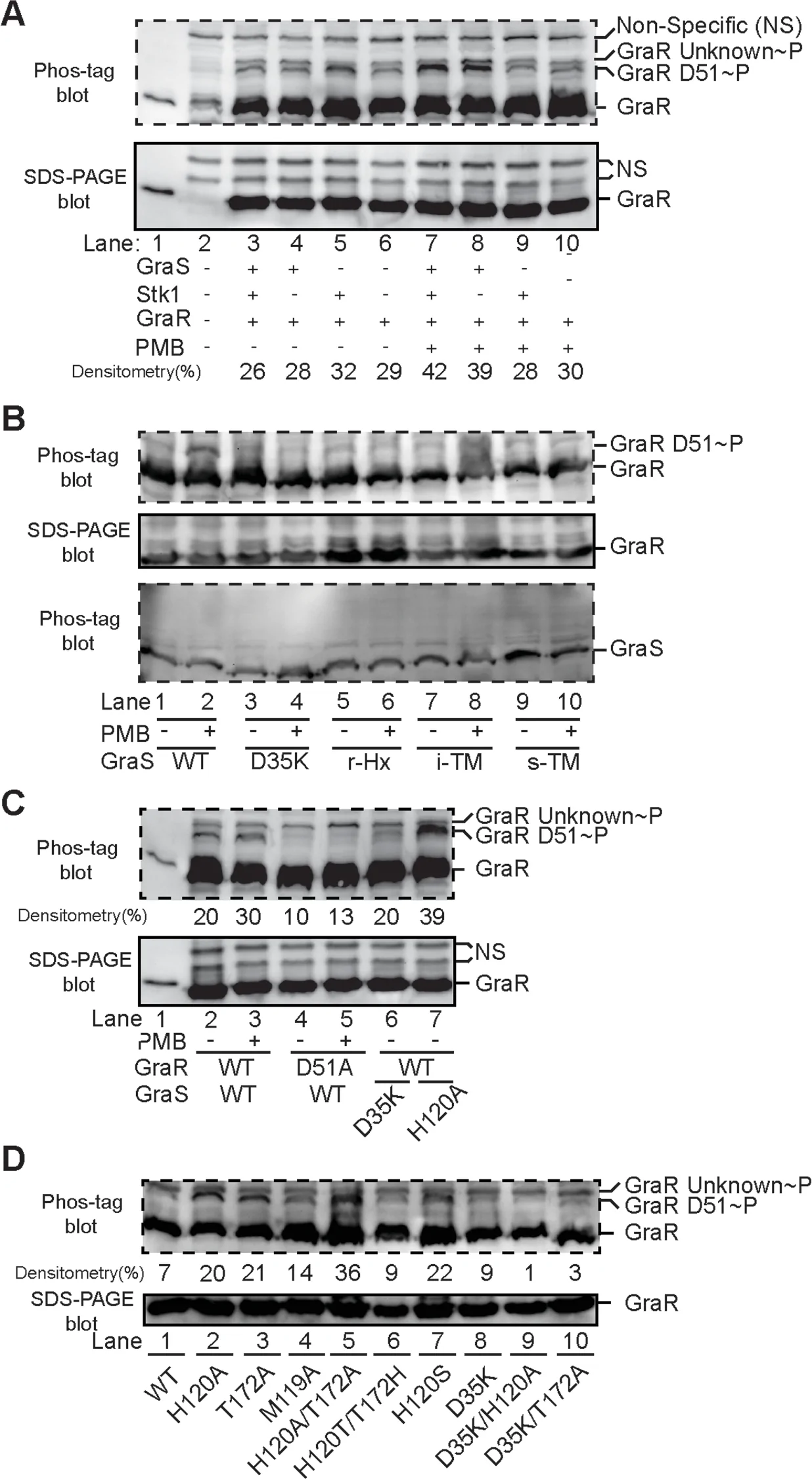
Phosphorylation assay of GraR in assorted *graS* mutants. *S. aureus* cells were lysed and analyzed by Phos-tag and standard SDS-PAGE gels. The purified HA-tagged GraR was visualized in Western blotting with anti-HA antibody (Fig. S10 in the supplemental material). (**A**) Phosphorylation of *graR* in the presence or absence of *graS* or *stk1*. Lane 1: purified GraR with HA-tag, lane 2: JE2 Δ*graXRS* Δ*stk1*/pEPSA5::blank, lane 3: JE2 Δ*graXRS*/pEPSA5::*graXRS* with HA-tag, lane 4: JE2 Δ*graXRS* Δ*stk1*/pEPSA5::*graXRS* with HA-tag, lane 5: JE2 Δ*graXRS*/pEPSA5::*graXR* with HA-tag, lane 6: JE2 Δ*graXRS* Δ*stk1*/pEPSA5::*graXR* with HA-tag, and lanes 7–10 represent samples that were treated with 64 mg/mL PMB for 30 min before harvesting. The dotted rectangle represents the blot from Phos-tag SDS-PAGE, and the solid box is from a standard SDS-PAGE. GraR and GraR-P are labeled next to the panel. (**B**) Phosphorylation of GraR in selective *graS* mutants. All samples with various *graS* mutations were constructed from JE2 Δ*graXRS* Δ*stk1*/pEPSA5::*graXRS* with HA-tag. The top panel shows GraR/GraR~P proteins on an immunoblot of Phos-tag gel detected with anti-HA antibodies, while the middle panel depicts an immunoblot of SDS-PAGE detected with anti-HA antibodies. The bottom panel exhibits a Phos-tag immunoblot probed with anti-GraS antibody. The even lanes represent samples treated with PMB. (**C) and (D**) Phos-tag immunoblots with anti-HA antibodies (top panel) for GraR and GraR-P proteins and SDS-PAGE (lower panel) of GraR in *graR* D51A mutant, *graS* D35K, and *graS* H120A mutants in panel C, while assorted *graS* mutant with D35K and/or H120A and/or T172A mutations as well as *graS* M119A (control) and *graS* H120S mutants are in panel D. The data have been verified in repeated and independent experiments. Densitometry = 100 * (GraR~ P/GraR in the standard SDS-PAGE). The densitometry values were calculated from a representative figure due to significant errors of standard deviation when analyzed from several distinct experiments. However, the phosphorylation trends were observed to be similar.

To avoid the issue of background phosphorylation, we first deleted the phosphorylase *stk1*, which has the capacity to phosphorylate GraR (21). For this purpose, we constructed JE2 Δ*graXRS* Δ*stk1*. To normalize the quantities of phosphorylated GraR, we measured the densitometry of GraR-P and total GraR in SDS-PAGE, and then calculated the ratio of GraR-P/GraR. As a negative control, the bands observed in lane 2 of Fig. 3A were considered non-specific bands. The band located above GraR D51-P, which was predicted to lack GraS-mediated phosphorylation, likely depicted phosphorylated-GraR on serine or threonine residues, or other phosphorylation events by unknown kinases. However, we observed no significant changes in this phosphorylation pattern with or without the treatment of PMB. Upon treatment with PMB, the amounts of the phosphorylated GraR in all strains with intact *graS* were increased, regardless of the status of *stk1* (Fig. 3A, lanes 3 and 4 vs 7 and 8). However, in strains lacking *graS*, the phosphorylation effects of GraR with PMB disappeared (Fig. 3A, lane 5 vs 9). These results indicate that GraS is the primary sensor to transduce the signal from PMB, leading to GraR phosphorylation.

We next tried to verify if EL and TM mutations in GraS impaired the phosphorylation of GraR with PMB. As expected, treatment with PMB increased phosphorylation of GraR in the parental strain JE2 (Fig. 3B, lanes 1 and 2). Importantly, phosphorylation of GraR was neither detected in the D35 mutant nor in the TM mutants (r-Hx, i-TM, and s-TM). Moreover, PMB treatment did not alter phosphorylation of GraR in these mutants with reduced *mprF* expression (Fig. 3B, first panel, lanes 3 and 4), corresponding to our previous results (Fig. 1). Besides GraR, we also attempted to visualize GraS phosphorylation, which is a prerequisite step for phosphor-relay to GraR; unfortunately, the phosphorylated form of GraS was not readily detectable in the Phos-tag gel (Fig. 3B, third panel), perhaps due to its transient nature and/or instability.

The aspartate residue in GraR at position 51 (D51) is the putative site of phosphor-transfer from GraS to GraR (18). Hence, we checked whether the *graR* D51A mutant could obtain a phosphor-group upon PMB treatment. As shown in Fig. 3C, the *graR* D51 mutant disclosed weak, if any, phosphorylated bands. These results reaffirmed that D51 in GraR is the residue that accepts the phosphor-group in GraRS TCS.

Consistent with the results of *mprF* expression (Fig. 2B and C), the mutations in the DHp domain (GraS H120A, T172A, or H120A/T172A) increased the levels of the phosphorylated GraR (Fig. 3C and D), especially with H120A/T172A double mutant, consistent with the *mprF* expression data. In the H120S mutant, which is predicted to form hydrogen bond with the threonine at position 172, phosphorylated GraR in the H120S mutant appeared to be increased in a reproducible manner, indicating that the mutation likely mildly lifted the restraint of rotational movement of the DHp domain as compared to the H120A or T172A mutant, but not as much as the H120A/T172A double mutant (Fig. 3D). Consistent with the idea that H120 interacts with T172, swapping the two residues (H120T/T172H) yielded comparable levels of GraR phosphorylation as the parental strain (Fig. 3D, lane 6). Importantly, in the presence of the D35K mutation, the H120A/T172A double mutant lost the augmented effect on phosphorylation of GraR (Fig. 3D, lanes 8–10). The *graR* M119A mutant (a negative control with M119 adjacent to H120) had the equivalent amount of phosphorylated GraR as the wild type (Fig. 3D, lane 4). Due to technical difficulties associated with using Phos-tag with whole cell lysate, obtaining a clear visualization of GraR-P bands in the Phos-tag gel resulted in blurring in the SDS-PAGE gel (Fig. 3D), as both membranes were developed simultaneously. Despite this challenge, we were able to observe similar phosphorylation trends in GraR across multiple biological replicates. These results indicate that D35 is the primary and necessary residue within the EL of GraS necessary for phosphor-relay from GraS to GraR, while H120 and T172, two residues within the cytoplasmic helices of GraS, modulate GraS activity but cannot override the effect of D35.

## DISCUSSION

Histidine kinases anchored in cell membranes generally sense environmental stimuli to activate the response regulators in bacterial TCS (4). However, a subset of bacterial TCS mostly in Firmicutes, designated as intramembrane histidine kinases, contains membrane-anchored HKs with a small EL (<10 amino acids) and is linked to an adjacent ABC transporter system (e.g., *graRS-vraFG*). Recent data suggested that GraS, the HK sensing HDPs, positively regulates expression of the adjacent *vraFG* for sensing cationic HDPs, while the 200-residue EL of the membrane permease VraG interferes with the sensing of HDPs by GraS (5). Although the state of GraS in the absence of CAMPs remains unknown, the GraRS regulons (MprF, DltABCD, and VraGF) are known to be constitutively expressed (18, 21, 22). Remarkably, the GraS protein contains three negatively charged residues (D35, D37, and D41) in the 9-residue EL, where cationic HDPs likely interact. Among the three aspartic residues within the EL of GraS, we showed that D35 is the critical residue that controls the sensory activity of GraS. Notably, sublethal concentrations of PMB (2 µg/mL) did not induce activation of the GraRS TCS in both the *graS* D35K and Δ*graS* mutants (Fig. S1 in the supplemental material), but it did with the parental strain. In our previous paper, we reported that the minimum inhibitory concentration (MIC) of PMB for the wild type was ~128 µg/mL, whereas it was in the range of 4–16 μg/mL for the Δ*graS* mutant. However, at a higher concentration (32 µg/mL), there was an increase in *mprF* expression even in the Δ*graS* mutant, as observed in the qRT-PCR analysis (Fig. S8 in the supplemental material). It is important to mention that the data obtained at this higher concentration should be interpreted with caution due to potential abnormal housekeeping gene expression during cell death. Based on molecular modeling, D35 is predicted to be at the junction between the EL and the first transmembrane segment (TM1), we surmise that the mutation at D35 (D35K) most likely influences its adjacent conformation (TM1) and, ultimately, the interaction of TM1 with TM2 (see Fig. 1) due to its charge alterations near the membrane surface (from negative to neutral or positive). Accordingly, we surmise the signal transduction of GraS must entail alteration in membrane (TM1 or with TM2) conformation, leading to secondary effect(s) on phosphorylation of H129 in GraS in the cytosol. This effect on GraS signaling appears to be specific because swapping TM1 and TM2 or inverting TM1 or removing the N-terminal helix yielded low level of *mprF* expression and a failure to phosphorylate GraR, similar to what has been observed in the D35 mutant (Fig. 1F and 3B). Besides the position of D35 near the TM1, the negative charge is also critical since mutation from aspartic acid (D) to glutamic acid (E) retained the signal activity of GraS while altering the charge disrupted GraS-mediated sensing, suggesting that the negative charge may hold a repulsive interaction with the negatively charged bacterial membrane head groups, leading to a fine-tuned TM1/2 conformation. In addition, mutation at the D41 residue, which is located near the other end of the loop (Fig. S2 in the supplemental material), also showed reduced GraS signaling activity, but less than that of D35K, hinting that both ends of the loop likely play a vital role in maintaining the TM1/TM2 conformation. To further investigate the effects of the D35 mutation in GraS activity, we attempted to examine the interaction between GraR and GraS with bacterial two hybrid system. However, the interaction between GraR and GraS was too weak to yield any meaningful data (Fig. S7 in the supplemental material). This failure may be due to the strict interaction requirement of the phosphorylated species. Alternatively, the interaction may require the presence of scaffolding protein such as GraX, given that we can only purify GraR and GraS in the presence of GraX.

Most HKs are generally known to be constitutively homodimeric (9, 23) but there are cases where ligand binding is required to induce dimer formation (10). Upon binding to environmental stimuli, HKs undergo their own conformational changes in the DHp domain, leading to *cis* or *trans* autophosphorylation. This autophosphorylation process is inversely related to the dissociation of the dimer into inactive monomers (24). In our study, we were able to identify the dimeric state of GraS with BACTH analysis in the absence of stimuli, which aligns with the previously reported data (18). This observation strongly suggests that GraS histidine kinase most likely exists in a dimeric form, following a typical histidine kinase model. Previous *in vitro* studies have mostly focused on HK activities from truncated cytoplasmic domains. In the case of full-length HKs, prior investigations have focused on kinase activities in the presence of external stimuli (10). Thus, the effect of HK dimerization on signaling activity, especially in IM-HK where the EL is extremely short, has not been fully evaluated. Our study showed that the D35K mutation within the EL of GraS impaired signaling activity (i.e., *mprF* expression) without affecting dimerization. We consider several scenarios for this observation: (i) the mutation may alter the conformation of the transmembrane domains, inhibiting interaction with GraR and (ii) the mutation may interfere with the ability of GraS to form a proper autophosphorylation conformation. Both assumptions converge on the observation that the sensor domain involving the EL and transmembrane segments in HKs participates in HK activation, especially in IM-HKs.

Recent studies have focused on piston-lever-like/rotational movements of the HAMP and DHp domains in HKs as a mechanism for signal transduction whereby increased rotation translates to activation (16, 25). With sequence alignment, we pinpointed the residues homologous to the ones reported in BceS. The mutations of the two residues (H120A and T172A) in GraS clearly enhanced signal transduction, and the double H120A/T172A mutant revealed higher activation (Fig. 2). However, the impact of the D35 mutation on activation was so crucial that even the introduction of H120A, T172A, or double H120A/T172A mutation could not counteract its down-regulatory effects on GraS signaling activity (Fig. 2; Fig. S3 in the supplemental material). Similar to the D35K mutation, the H120A and T172A mutations did not affect dimerization (Fig. 2F). These results strongly suggest that intracellular signal transduction from the DHp domain is important for the activation of histidine kinases.

We also explored if hydrogen bonding between H120 and T172 could account for the interaction that stabilizes the two helices within the DHp domain of GraS. Accordingly, the *graS* H120T/T172H mutant was used to reaffirm whether the two residues (H120 and T172) act as a hinge in the helical rotation of DHp domain. Florescence assay with the *mprF* promoter in the double H120T/T172H mutant yielded similar activity as the wild-type JE2 but had slightly higher *mprF* expression in the qRT-PCR assay, meaning that the higher sensitivity of the qRT-PCR assay may have revealed a slight lift for the activation in the double mutant vs the parental strain (Fig. 2). Likewise, a H120S substitution, allowing hydrogen bonding with T172, had the same effect as the double H120T/T172H substitution but higher than the parental strain. We speculate that the *graS* H120S mutation might attenuate the steric hindrance by replacing histidine’s bulky imidazole ring with the serine’s hydroxyl group, leading to improved rotation of the DHp domain. Together, these studies confirm that interaction between H120 and T172 in GraS likely occurs via hydrogen bonding to stabilize the helices TM1 and TM2. Disruption of the bonding would facilitate more rotation to enable the proper conformational change for signal transduction.

While we have been focusing on GraS signaling for the TCS, a group looked at a universal serine/threonine kinase (Stk1 aka PknB), which phosphorylates three threonine residues in GraR, suggesting crosstalk between GraRS TCS and Stk1/Stp1 (21). We, however, needed further information to evaluate if the phosphorylation of GraR by Stk1 without the aid of GraS impacted GraRS TCS as an additional protective mechanism against CAMPs. Here, we show that GraS alone responded to the signal from cationic antibiotic (PMB) and phospho-relayed onto GraR, in a Stk1-independent manner (Fig. 3A).

**Fig 4:**
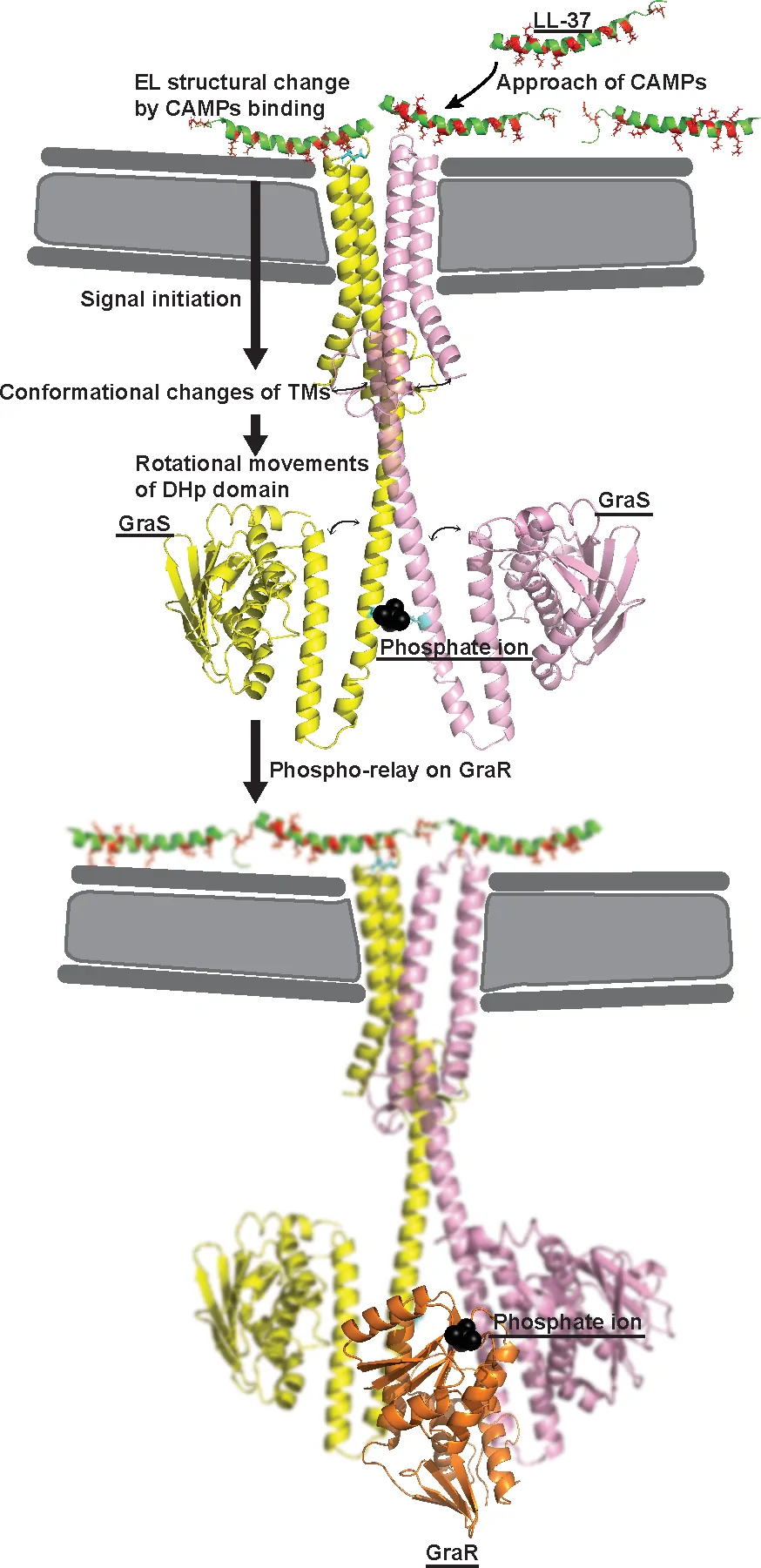
A proposed model of GraS signaling. Mutations in the DHp domain activate GraRS TCS. The modeled GraS and GraR (12) and a crystal structure of LL-37 [PDB ID: 2K6O (27)] were used to illustrate the signaling mechanism of GraS. When LL-37 binds the EL of GraS, their interaction may induce changes in the conformation of TMs and the HAMP domain, forwarding the signal onto the helices in the DHp domain. If the rotational movement of the cytoplasmic helices, normally restricted by the H120-T172 interaction, occurs, autophosphorylation would be rapidly attained and eventually relay the phospho-group to GraR.

Considering histidine kinases in TCSs and IM-HK, in particular, there seems to be three major domains involved in signal transduction: EL, transmembrane, and cytosolic domains which include HAMP, DHp, and ATPase domains. Previously, the cytosolic domains have been used in *in vitro* analysis for autophosphorylation and dimerization study, primarily due to the solubility and expression/purification issues with full-size membrane proteins (26). Using GraXRS as a platform for expression, we were able to purify enough GraS for our study, which reveals the importance of D35 in EL and the membrane constituents TM1 and TM2 in the signal transduction of GraRS. Considering that D35 is a critical residue in the EL that drives GraS-mediated signaling upon contact with cationic HDPs (3) and that H120A and T172A cannot overcome the signaling defect in a D35K, we propose that the signal moves from D35 and D41 in the EL to TMs, HAMP, and DHp through a stepwise process (Fig. 4). The binding of HDPs to EL via D35 and D41 likely initiates the process, leading to conformational changes in TM helices, followed by signaling to HAMP and moderating activity via the helical rotation of the DHp domain. This process may be facilitated by H120 and T172 in the DHp domain. Our studies suggest that H120 likely forms hydrogen bond with T172 to stabilize the helices and disruption of the helices in the DHp domain likely increases the rotation of the helices to facilitate exposure of H129 in GraS for phosphorylation. Importantly, this process did not affect dimerization of GraS. In the absence of CAMPs, it is likely that GraS has a basal level of activation via phosphorylation and transfer of a phosphor-group to GraR to maintain a basal positive membrane charge. We anticipate that this study contributes to understanding the signaling mechanism for TCS and underlining the significance of EL and TMs in IM-HK. Whether this model of activation applies to TCS systems other than IM-HK remains to be determined.

## MATERIALS AND METHODS

### Bacterial strains and materials

*Staphylococcus aureus* and *Escherichia coli* bacterial cells were grown in tryptic soy broth (TSB, BD Bacto) and cation-adjusted Mueller-Hinton broth (CAMHB, Mueller-Hinton broth with 25 mg/L Ca^2+^ and 12.5 mg/L Mg^2+^, BD Difco) or LB media, respectively. Lysogeny broth (LB) and TSB were autoclaved at 121°C for 20 min, and CAMHB was sterile filtered with 0.22 μm pore size filter. *E. coli* IM08B was used to restriction-modify plasmids for direct transformation into MRSA strain JE2 (wild type) (28, 29). When necessary, proper antibiotics were added to the media: ampicillin (100 μg/mL), chloramphenicol (10 μg/mL), or erythromycin (2.5 μg/mL). If not mentioned, cells were grown at 37°C at 250 rpm in a reciprocating shaker. Overnight cultures were grown in 5 mL media. The flask volumes were adjusted to five times the culture volume (e.g., 50 mL flask for 10 mL culture). The specific culture volumes used for each method were described in their respective sections.

### Chromosomal mutations and plasmid construction

Allelic replacement was performed with a pMAD vector for bacterial chromosomal mutations (13). The competent cells prepared with 10% glycerol were resuspended in 10% glycerol and 500 mM sucrose, followed by transformation with recombinant pMAD vectors. After transformation with an electroporator (2.1 V in 0.1 cm cuvette, Bio-rad, a gene pulser), the cells were allowed to recover in 1 mL of TSB for 1 h and then spread on a 2.5 μg/mL erythromycin (erm)/80 μg/mL X-gal TSA plate followed by incubation at 30°C for 2 days. One blue colony was incubated in 5 mM TSB with 2.5 μg/mL erm overnight at 30°C. The following day, 50 μL of overnight culture was diluted in 5 mL of TSB without antibiotics and incubated at 43°C for 4 h. Diluted culture was plated on TSA with 2.5 μg/mL erm/50 μg/mL X-gal and again incubated at 43°C overnight. Three light blue colonies were selected, incubated in 5 mL of TSB without antibiotics at 30°C overnight, diluted and plated on TSA with X-gal and without antibiotics followed by incubation at 37°C overnight. White colonies were streaked on both erm/X-gal TSA and X-gal TSA plates. Mutants without the pMAD vector (no survival on the erm TSA plate but white streaks on the X-gal TSA plate) were selected and verified by PCR and DNA sequencing.

We adopted a general heat-shock method for *E. coli* transformation with plasmids by heating at 42°C for 30 s. To construct new plasmids with mutations (pMAD, pKT25, pKNT25, pUT18, pUT18C, and pEPSA5) (30), we used SOEing PCR followed by restriction enzyme cleavage (NEB) and T4 DNA ligation (NEB).

### GFP_uvr_ expression driven by *mprF* promoter

Plasmid pALC1484::*mprF* promoter (5) was transformed with JE2 wild type and mutants. The newly constructed strains were grown in 5 mL CAMHB overnight and diluted in 10 mL of CAMHB. After incubation for 3–4 h at 37°C, fluorescence (excitation: 487 nm and emission: 511 nm) and OD_600_ were measured using a spectrophotometer (BioPhotometer plus, Eppendorf) and a Tecan M1000 Pro plate reader (gain of 145).

### qRT-PCR analysis for *mprF* transcript

Cells grown in 10 mL TSB to the mid-log phase were treated with or without 32 μg/mL PMB at 37°C for an additional 30 min. Cells were harvested at 3,600 × *g* for 10 min, resuspended in 500 μL of Trizol and chilled in 2 mL screw cap tubes containing ~200 μL of silica/glass beads followed by bead-beater lysis (two times for 1-min shake and 1-min break in an ice-bucket). The lysed samples were mixed with 100 μL of chloroform and incubated at room temperature for 3 min. For RNA separation, samples were centrifuged at 12,000 *g* for 15 min at 4°C, and the aqueous phase supernatant was collected. To precipitate RNAs, 200 μL of isopropanol was added followed by incubation for 10 min at RT and centrifuged (12,000 *g* for 10 min at 4°C). The precipitate was washed with 500 μL of ice-cold 70% ethanol, spun, air-dried, and resuspended in 100 μL of nuclease-free water. For removal of DNA contamination, a 50 μL reaction (10 μg of RNA + 1 μL of turbo DNase + 5 μL of turbo DNase buffer) was performed at 37°C for 30 min followed by the DNAse inactivation process (Invitrogen, turbo DNA free kit). For cDNA synthesis (Thermo fisher, RevertAid H minus first strand cDNA synthesis kit), we made a 12 μL reaction (500 ng of total RNA + 1 μL of 20 μM specific primer) and incubated at 65°C for 5 min to properly denature and anneal the primers and cooled down at 4°C. The cDNA synthesis was conducted by following the manufacturer’s protocol by adding a reaction cocktail (4 μL of reaction buffer + 1 μL of Ribolock RNase inhibitor + 2 μL of 10 mM dNTP mix + 1 μL RevertAid H minus reverse transcriptase). The cDNA dilutants (×100) were analyzed with SsoFast master mix and iQ SYBR green supermix (Bio-rad), using Bio-rad CFX96. The primers used (Table S2) were designed in https://bioinfo.ut.ee/primer3-0.4.0/ with settings of 20 bp primer size and ~150 bp product size.

### Cytochrome c-binding assay

The cells in 10 mL of CAMHB (after 1:100 dilution) grown to an OD_650_ of 0.5 were spun at 3,600 × *g* for 10 min, washed with 1× 3-(N-morpholino) propanesulfonic acid (MOPS) twice (5,000 × *g* for 2 min), and resuspended in 700 μL of MOPS. To determine the cell density, we diluted the sample with 1× MOPS and then measured the sample at A_650_. The raw mixture was adjusted with the final concentration of 3 of A_650_. Five hundred microliters of the sample were spun at 5,000 × *g* for 2 min and resuspended in 500 μL of 0.25 mg/mL cytochrome c solution. The resuspension was incubated with a rotary shaker for 10 min at room temperature and centrifuged at 5,000 × *g* for 2 min. Two hundred microliters of the supernatant were removed and read on a 96-well plate at A530 using the Tecan M1000 Pro reader.

### LL-37 2-h killing assay

Cells were grown to the mid-log phase (10^8^ CFU/mL, OD_600_ ~ 0.5) in 10 mL of brain heart infusion (BHI) at 37°C. The cells were diluted to 10^6^ CFU/mL in 10 mM KH_2_PO_4_ (PPB) + 1% BHI. We prepared the reaction mixture by mixing 160 μL of 10 mM PPB + 1% BHI, 20 μL of the diluted cells, and 20 μL of 25 μg/mL LL-37. As a control, a sample was prepared without LL-37, diluted to 10^3^ CFU/mL, and spread on a TSA plate. The sample with LL-37 was incubated at 37°C for 2 h, diluted to 10^3^ CFU/mL, and spread on a TSA plate, followed by incubation at 37°C overnight. The following day, we counted the number of colonies and calculated survival percentages based on them.

### BACTH assay

DHT1 was co-transformed with pUT18 (pUT18C) and pKT25 (pKNT25) plasmids and grown on LBA with 50 μg/mL kanamycin, 100 μg/mL ampicillin, 0.5 mM IPTG, and 40 μg/mL X-gal followed by incubation at 30°C for 2 days (18). We picked blue-color colonies and grew them overnight in 5 mL LB with kanamycin and ampicillin plus 0.5 mM of IPTG at 30°C. The overnight culture was diluted in M63 medium (1 mL cells + 4 mL 1× M63), and OD600 was measured from each sample. Aliquot of 1.25 mL in a 14 mL Falcon tube was treated with 25 μL chloroform and 25 μL 0.1% SDS to permeabilize the cells. After 30-min incubation at RT, a 32 μL aliquot was added to 128 μL of PM2 (70 mM Na_2_HPO_4_·12H_2_O, 30 mM NaH_2_PO4·H_2_O, 1 mM MgSO_4_, 0.2 mM MnSO_4_, pH 7.0, and 100 mM β-mercaptoethanol) in a 96-well plate. Forty microliters of 0.4% ONPG in PM2 without β-mercaptoethanol were added to the mixture to initiate the reaction (the final volume was 200 μL). We measured β-galactosidase activities at 420 nm (a fixed setting for GraS dimerization, 10-min incubation at room temperature under a dark environment) using the Tecan reader. The final values were calculated by


$$200\;\times \left(the\;dilution\;factor\right)\;\times \;\frac{A420\;of\;sample\;-\;A420\;of\;control}{OD600}$$

### Phosphorylation assay and Western blotting

*S. aureus* cells grown in 100 mL CAMHB or TSB to mid-log phase were treated with or without 64 μg/mL of PMB for 30 min. The cells were washed, resuspended in 10 mL of TBS (50mM Tris-HCl, 150mM NaCl, pH 7.4), dehydrated with 10 mL of ethanol/acetone (1:1) (31, 32), and incubated at 4°C for 5 min, followed by washing with TBS and resuspending in 100 μL of 50 mM Tris, pH 7.4. The resuspension was lyzed with bead-beating three times (30 s on/30 s break in an ice bucket), spun at 12,000 *g* for 15 min at 4°C, and measured at A_595_ with Bradford assay. Ten micrograms of the lysate were dissolved in a 4× SDS-PAGE sample buffer (0.2 mg/mL bromophenol blue, 250 mM Tris-Cl, pH 6.8, 8% SDS, 40% glycerol, and 20% of 14.3 M 2-Mercaptoethanol) without EDTA (final volume of 10 μL in 1× buffer), incubated at room temperature for 15 min, and the sample was loaded on SDS-PAGE or Phos-tag SDS-PAGE gels. To retain phosphorylation on GraR, we excluded heat denaturation. For Phos-tag SDS-PAGE gel, we prepared 10% SDS-PAGE with 50 μM of Phos-tag and 100 μM of MnCl^_2_^. The gel was run at 70 V at RT until reaching the resolving gel (for ~15 min) and the voltage was increased to 150 V (for ~1 h 40 min). For western blotting with HA antibody, the gel was incubated in 100 mL of transfer buffer (25 mM Tris, pH 8.3, 190 mM glycine, 20% Methanol, and 10 mM EDTA) for 15 min, washed with the transfer buffer without EDTA, and transferred to a PVDF membrane using iBlot2 (Invitrogen). The membrane was blocked with 5% BSA in TSBT at room temperature for 1 h and then incubated with a primary antibody [HA antibody (Abcam, ab18181) at 1:1,000 dilution to detect GraR, or GraS antibody (11) at 1:1,000] in 5% BSA followed by incubation at 4°C overnight. The following day, the membrane was washed with TSBT and incubated with a secondary HRP-conjugated goat anti-mouse antibody at 1:5,000 dilution in 5% BSA for 1 h. After washing with TBST thrice, the membrane was agitated with Pierce ECL western blotting substrates for chemiluminescence and visualized with the ChemiDoc MP imaging system (Bio-rad). For western blotting with NBT/BCIP, the blotted membrane was washed in blocking buffer (10 mM Tris pH 7.5, 500 mM NaCl, 0.5% Tween 20, and 0.04% sodium azide) for 1 h at room temperature in a gentle shaker followed by incubation with a primary antibody [GraS antibody 1:1,000 dilution (9)] at room temperature for 2 h. The membrane was washed with blocking buffer thrice and incubated with a secondary antibody (alkaline-phosphatase-conjugated anti-Mouse IgG, F(ab′)_2_, 115-056-072, Jackson ImmunoResearch) for 1 h. To visualize the bands, the membrane was again incubated with detection buffer (0.1 M Tris pH 9.5, 0.1 M NaCl, and 50 mM MgCl_2_) for 5 min and developed with 1-step BCIP/NBT solution (Ref. 34042, ThermoScientific). After rinsing with water, we digitalized the membrane picture with a scanner (EPSON V700 photo).

## SUPPLEMENTAL MATERIAL

Supplemental material spectrum.01982-23-s0001.docx Fig. S1 to S10; Tables S1 and S2.

ASM does not own the copyrights to Supplemental Material that may be linked to, or accessed through, an article. The authors have granted ASM a non-exclusive, world-wide license to publish the Supplemental Material files. Please contact the corresponding author directly for reuse.
