# Supplementary material for: GraS signaling in Staphylococcus aureus is regulated by a single D35 residue in the extracellular loop
Source: Microbiol Spectr. 2023 Sep 20;11(5):e01982-23. doi: 10.1128/spectrum.01982-23 (PMC10581149; doi:10.1128/spectrum.01982-23)
Supplement: Supplemental material — Fig. S1 to S10; Tables S1 and S2. [file spectrum.01982-23-s0001.docx]

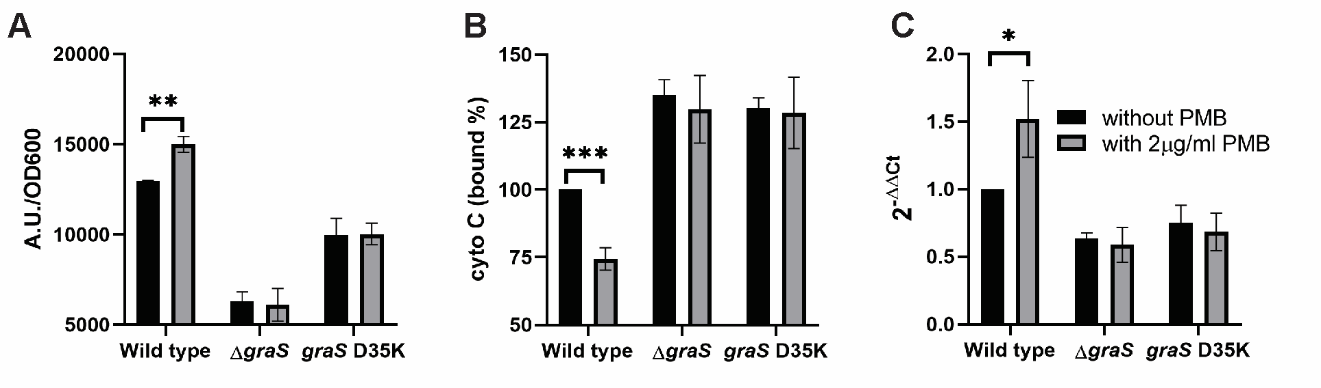


Fig. S1. GraRS TCS in graS mutants in the presence and absence of sublethal concentration of PMB. (A) and (C) *mprF* expression through GFP_uvr_ report and qRT-PCR. We utilized the ΔgraS mutant from our previous paper (5). (B) Cytochrome C binding. The sublethal concentration of PMB was used 2μg/ml. The stress was applied for 30 min when OD_600_ reached about 0.7. The values obtained from a minimum of three biological replicates were analyzed using a two-tailed Student's t-test, assuming equal variances. (*** 0.0001 to 0.001, ** 0.001 to 0.01, * 0.01 to 0.05, not labeled considered non-significant with p ≥ 0.05)


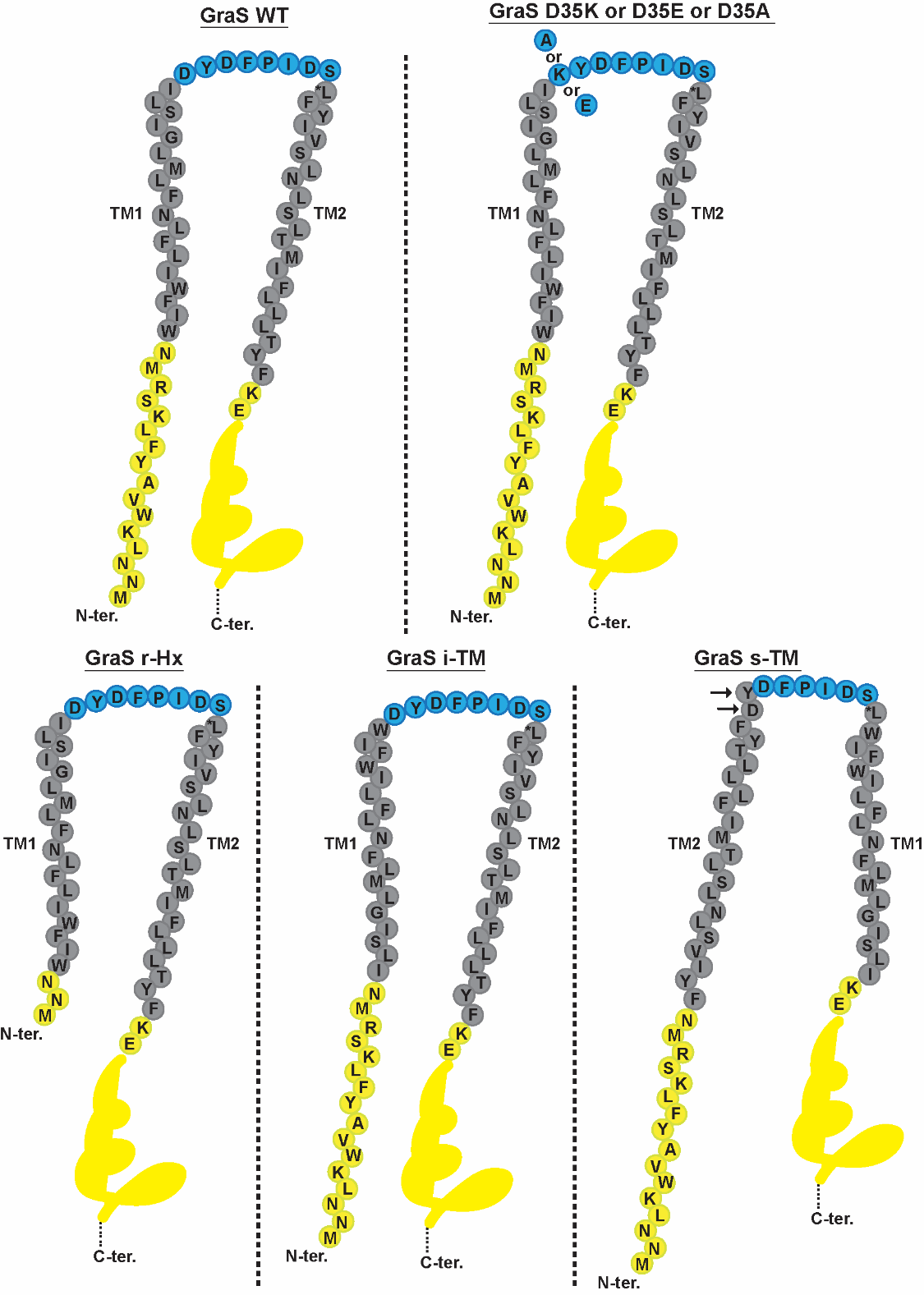


Fig. S2. Illustrated structures of GraS mutants. The residues were classified into three kinds (cytosolic portion: yellow, TM: gray, EL: blue). The transmembrane structures were predicted by DeepTMHMM (1).

1. J. Hallgren, et al., DeepTMHMM predicts alpha and beta transmembrane proteins using deep neural networks. 2022.04.08.487609 (2022).


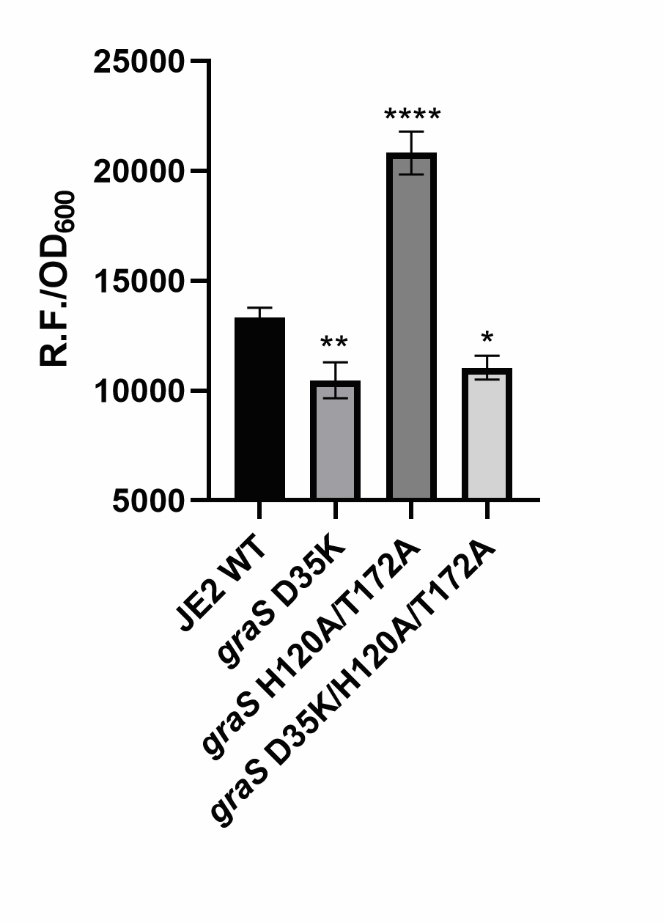


Fig. S3. GFP_uvr_ expression driven by mprF promoter. The values from at least three biological replicates were analyzed using one-way ANOVA vs the wild type. (**** p < 0.0001, *** 0.0001 to 0.001, ** 0.001 to 0.01, * 0.01 to 0.05)


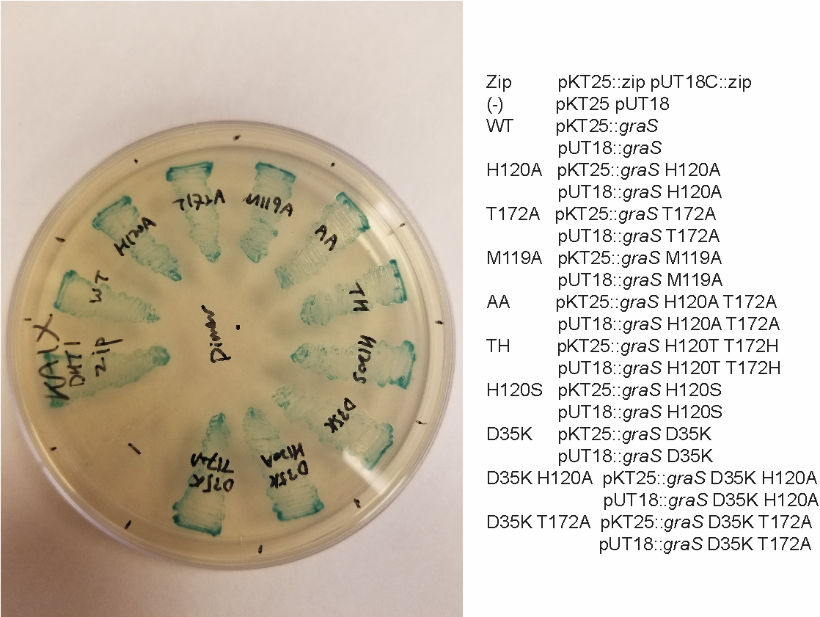


Fig. S4. GraS dimerization. BACTH assay was performed with DHT1 pKT25/pUT18 plasmid. The left panel shows the blue-colored streaks from all the samples on 40 mg/ml X-gal/0.5mM IPTG LB agar plates, indicating a strong interaction between two GraS proteins (dimerization).


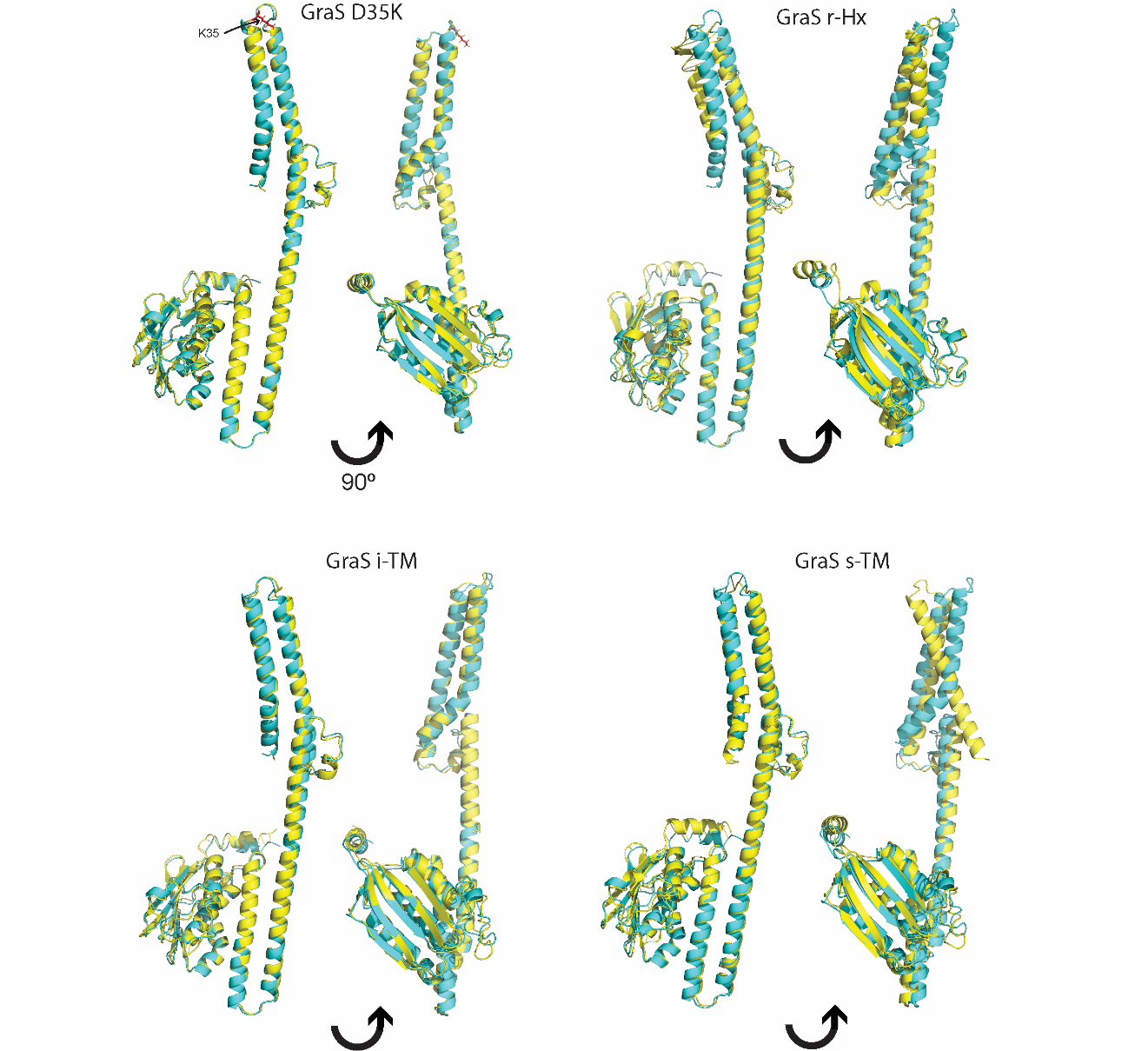


Fig. S5. Modeled structures of GraS mutants. Wild type GraS was colored cyan, while the mutants were shaded yellow.


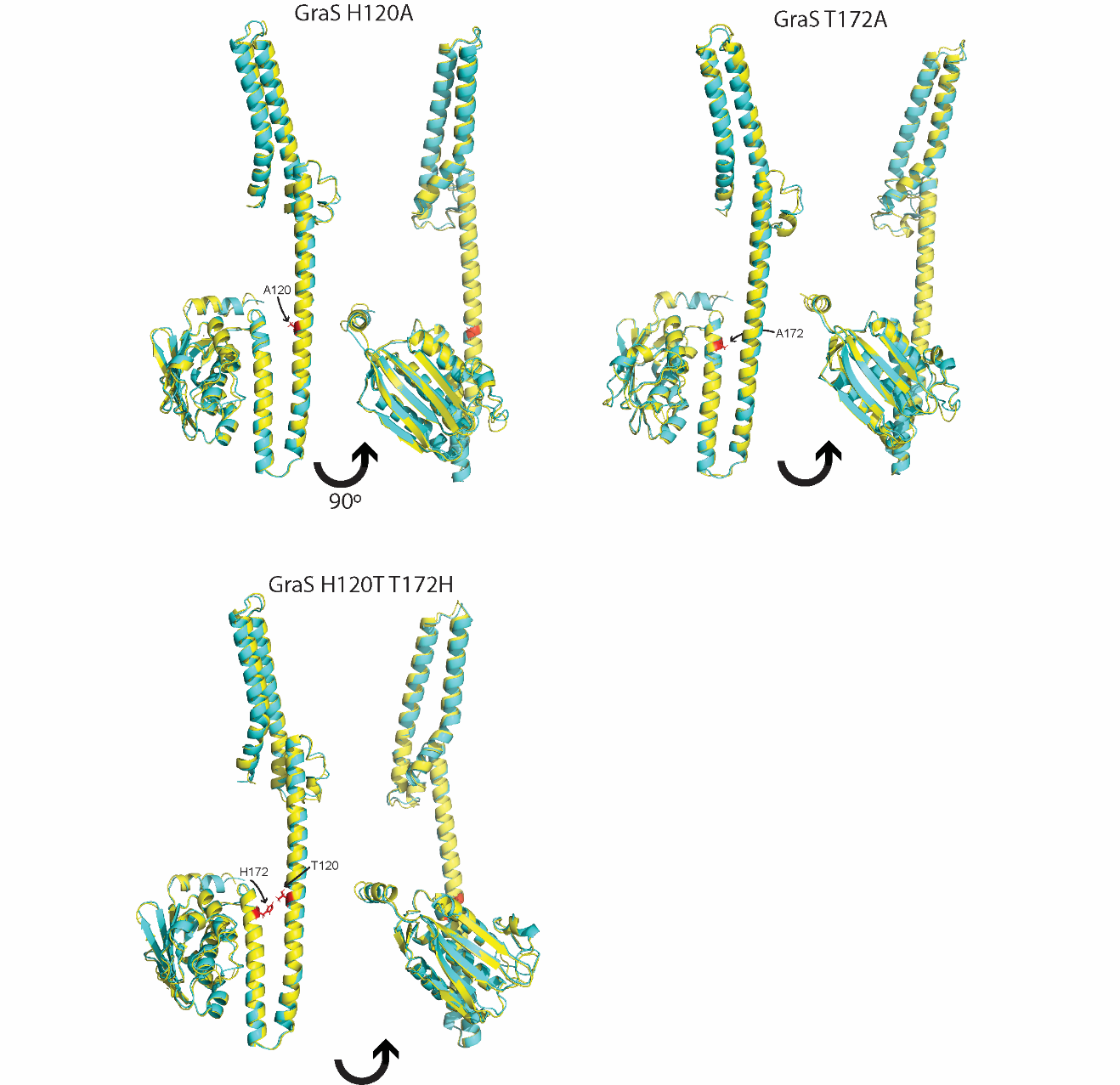


Fig. S6. Modeled structures of GraS mutants. The mutations at residues 120 and 172 in GraS were depicted as sticks. Wild type and mutants were colored cyan and yellow, respectively.


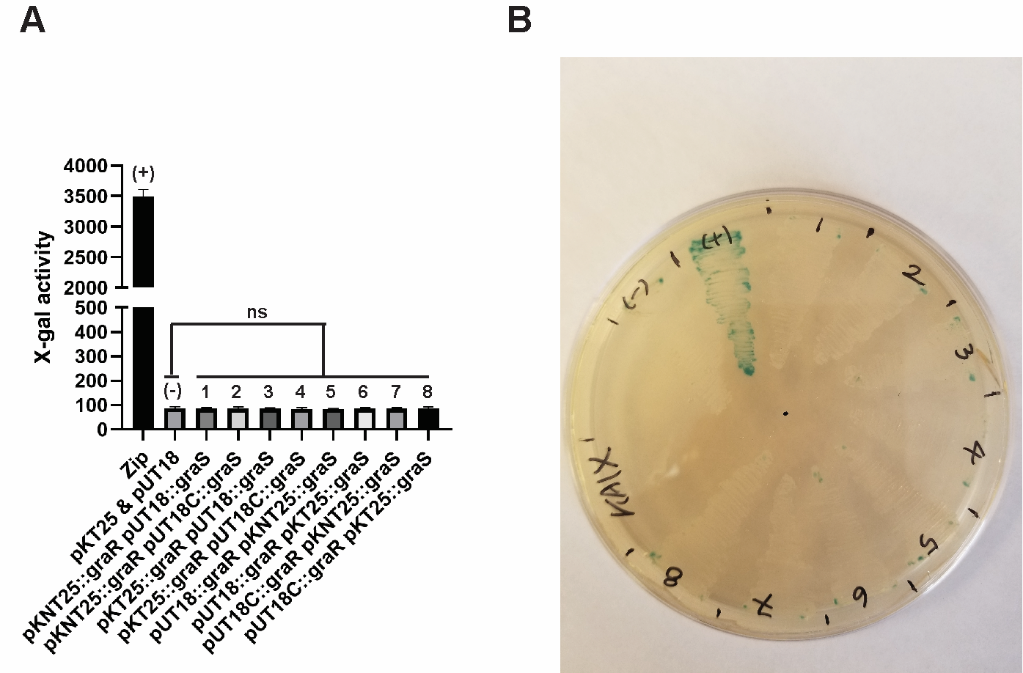


Fig. S7. BACTH assay for GraR and GraS interaction. The left panel (A) shows X-galactosidase activity with ONPG. (B) DHT1 strains with plasmids were incubated on X-gal / IPTG LB agar plates. The values from at least three biological replicates were analyzed using one-way ANOVA vs the wild type. (ns considered non-significant with p ≥ 0.05)


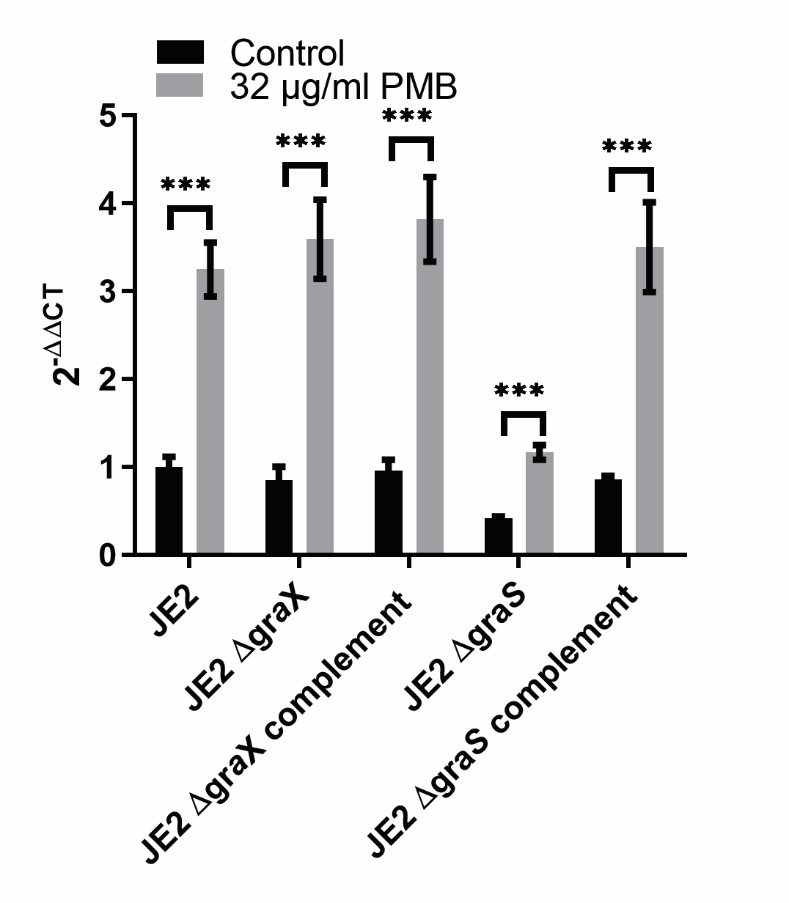


Fig. S8. qRT-PCR analysis for *mprF* transcript. We performed a two-tailed Student's t-test, assuming equal variances, to analyze the values obtained from three biological replicates. (**** p < 0.0001, *** 0.0001 to 0.001, ** 0.001 to 0.01, * 0.01 to 0.05)


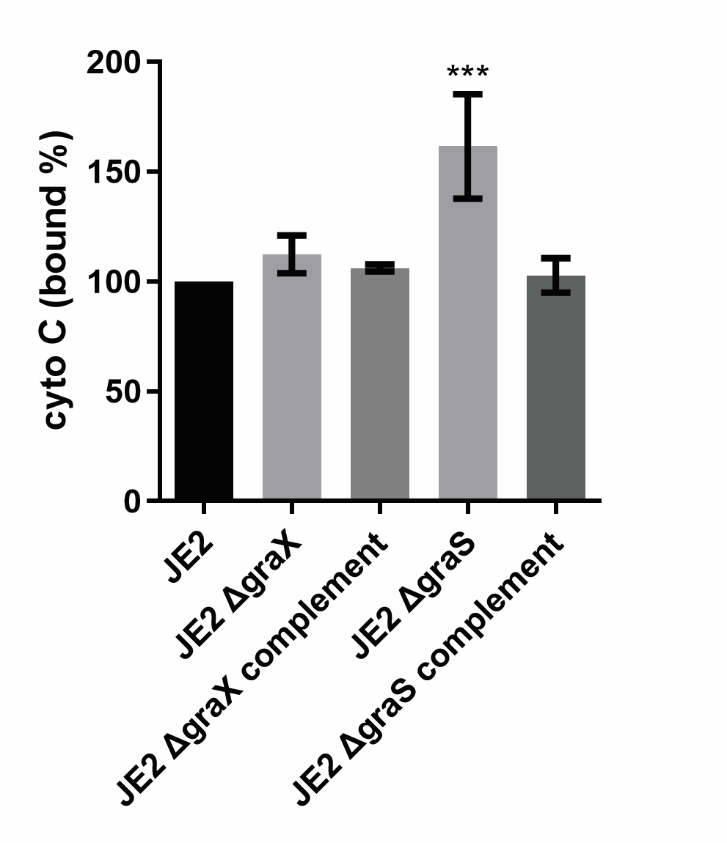


Fig. S9. Cytochrome c binding assay. The values from at least three biological replicates were analyzed using one-way ANOVA vs the wild type. (**** p < 0.0001, *** 0.0001 to 0.001, ** 0.001 to 0.01, * 0.01 to 0.05, not labeled considered non-significant with p ≥ 0.05)


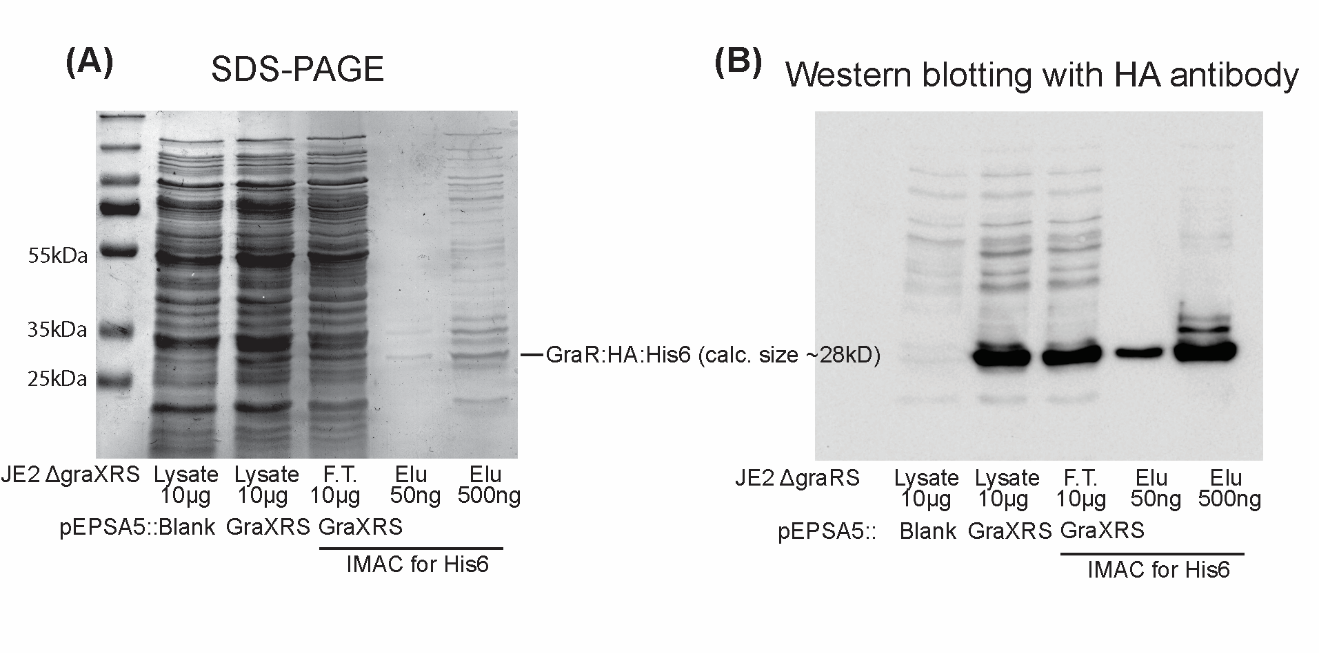


Fig. S10. Visualization of GraR. (A) SDS-PAGE. Lanes 1: Ladder, 2: Cell lysate of JE2 Δ*graXRS* pEPSA5::blank 3: Cell lysate of JE2 Δ*graXRS* pEPSA5::*graXRS* 4 and 5: Purified GraR with affinity chromatography. (B) Western blotting with HA antibody.

Table S1. Strains and Plasmids.

| Strains and plasmids | Features | Ref. |
| --- | --- | --- |
| JE2 (MRSA) | A derivative of USA300 LAC | (1) |
| JE2 *graS* D35K | Single mutation at *graS* D35K | This study |
| JE2 *graS* D35A | Single mutation at *graS* D35A | This study |
| JE2 *graS* D35E | Single mutation at *graS* D35E | This study |
| JE2 *graS* D37K | Single mutation at *graS* D37K | This study |
| JE2 *graS* D41K | Single mutation at *graS* D41K | This study |
| JE2 *graS* F38A | Single mutation at *graS* F38A | This study |
| JE2 *graS*  D35K/D37K/D41K | Triple mutation at *graS* D35K/D37K/D41K | (2) |
| JE2 Δ*graS* | *graS* deletion mutation | (2) |
| JE2 *graS* r-Hx | A strain encoding ΔN-terminal of GraS | This study |
| JE2 *graS* i-TM | A strain encoding inverted TMs of GraS | This study |
| JE2 *graS* s-TM | A strain encoding swapped TMs of GraS | This study |
| JE2 *graS* D35K complement | Chromosomal complementation from each mutant | This study |
| JE2 *graS* r-Hx complement |  |  |
| JE2 *graS* i-TM complement |  |  |
| JE2 *graS* s-TM complement |  |  |
| JE2 ΔEL vraG | EL deletion mutation in *vraG* | (2) |
| JE2 ΔEL vraG & *graS* D35K | ΔEL *vraG* with single mutation at *graS* D35K | This study |
| JE2 ΔEL vraG & *graS* D37K | ΔEL *vraG* with single mutation at *graS* D37K | This study |
| JE2 ΔEL vraG & *graS* D41K | ΔEL *vraG* with single mutation at *graS* D41K | This study |
| JE2 ΔEL vraG & *graS* F38A | ΔEL *vraG* with single mutation at *graS* F38A | This study |
| JE2 ΔEL vraG  *graS* D35K/D37K/D41K | ΔEL *vraG* with triple mutation  at *graS* D35K/D37K/D41K | This study |
| JE2 *graS* H120A | Single mutation at *graS* H120A | This study |
| JE2 *graS* T172A | Single mutation at *graS* T172A | This study |
| JE2 *graS* M119A | Single mutation at *graS* M119A | This study |
| JE2 *graS* H120A/T172A | Double mutation at *graS* H120A and T172A | This study |
| JE2 *graS* H120T/T172H | Double mutation at *graS* H120T and T172H | This study |
| JE2 *graS* H120S | Single mutation at *graS* H120S | This study |
| JE2 *graS* D35K/H120A | Double mutation at *graS* D35K and H120A | This study |
| JE2 *graS* D35K/T172A | Double mutation at *graS* D35K and T172A | This study |
| JE2 *graS*  D35K/H120A/T172A | Triple mutation at *graS* D35K, H120A and T172A | This study |
| pMAD | A plasmid for allelic recombination | (3) |
| pMAD *graS* D35K | pMAD with ~2000bp *graS* D35K flanking gene | This study |
| pMAD *graS* D35A | pMAD with ~2000bp *graS* D35A flanking gene. | This study |
| pMAD *graS* D35E | pMAD with ~2000bp *graS* D35E flanking gene. | This study |
| pMAD *graS* D37K | pMAD with ~2000bp *graS* D37K flanking gene. | This study |
| pMAD *graS* D41K | pMAD with ~2000bp *graS* D41K flanking gene. | This study |
| pMAD *graS* F38A | pMAD with ~2000bp *graS* F38A flanking gene. | This study |
| pMAD *graS* r-Hx | pMAD with ~2000bp *graS* r-Hx flanking gene. | This study |
| pMAD *graS* i-TM | pMAD with ~2000bp *graS* i-TM flanking gene. | This study |
| pMAD *graS* s-TM | pMAD with ~2000bp *graS* s-TM flanking gene. | This study |
| pMAD *graS* complement | pMAD with ~2000bp WT *graS* flanking gene. | (2) |
| pMAD *graS* H120A | pMAD with ~2000bp *graS* H120A flanking gene. | This study |
| pMAD *graS* T172A | pMAD with ~2000bp *graS* T172A flanking gene. | This study |
| pMAD *graS* M119A | pMAD with ~2000bp *graS* M119A flanking gene. | This study |
| pMAD *graS* H120A/T172A | pMAD with ~2000bp *graS* H120A/T172A flanking gene. | This study |
| pMAD *graS* H120T/T172H | pMAD with ~2000bp *graS* H120T/T172H flanking gene. | This study |
| pMAD *graS* H120S | pMAD with ~2000bp *graS* H120S flanking gene. | This study |
| pMAD *graS* D35K/H120A | pMAD with ~2000bp *graS* D35K/H120A flanking gene. | This study |
| pMAD *graS* D35K/T172A | pMAD with ~2000bp *graS* D35K/T172A flanking gene. | This study |
| pMAD *graS*  D35K/H120A/T172A | pMAD with ~2000bp *graS* D35K/H120A/T172A flanking gene. | This study |
| pALC1484::*mprF* promoter | A plasmid expressing GFP_uvr_ driven by mprF promoter | (2) |
| DHT1 | *E. coli* strain (*cya* deficient) for BACTH | (4) |
| pKT25::*graS* | A plasmid expressing GraS with T25 |  |
| pUT18::*graS* | A plasmid expressing GraS with T18 |  |
| pKT25::*graS* H120A | A plasmid expressing GraS H120A with T25 | This study |
| pUT18::*graS* H120A | A plasmid expressing GraS H120A with T18 | This study |
| pKT25::*graS* T172A | A plasmid expressing GraS T172A with T25 | This study |
| pUT18::*graS* T172A | A plasmid expressing GraS T172A with T18 | This study |
| pKT25::*graS* M119A | A plasmid expressing GraS M119A with T25 | This study |
| pUT18::*graS* M119A | A plasmid expressing GraS M119A with T18 | This study |
| pKT25::*graS* H120A/T172A | A plasmid expressing GraS H120A/T172A with T25 | This study |
| pUT18::*graS* H120A/T172A | A plasmid expressing GraS H120A/T172A with T18 | This study |
| pKT25::*graS*  H120T/T172H | A plasmid expressing GraS H120T/T172H with T25 | This study |
| pUT18::*graS*  H120T/T172H | A plasmid expressing GraS H120T/T172H with T18 | This study |
| pKT25::*graS* H120S | A plasmid expressing GraS H120S with T25 | This study |
| pUT18::*graS* H120S | A plasmid expressing GraS H120S with T18 | This study |
| pKT25::*graS* D35K | A plasmid expressing GraS D35K with T25 | This study |
| pUT18::*graS* D35K | A plasmid expressing GraS D35K with T18 | This study |
| pKT25::*graS* D35K/H120A | A plasmid expressing GraS D35K/H120A with T25 | This study |
| pUT18::*graS* D35K/H120A | A plasmid expressing GraS D35K/H120A with T18 | This study |
| pKT25::*graS* D35K/T172A | A plasmid expressing GraS D35K/T172A with T25 | This study |
| pUT18::*graS* D35K/T172A | A plasmid expressing GraS D35K/T172A with T18 | This study |
| pKT25::zip | A plasmid expressing leucine zipper GCN4 domain | (5) |
| pUT18C::zip | A plasmid expressing leucine zipper GCN4 domain |  |
| pKT25 | A plasmid expressing a target gene on the C-terminal with T25 |  |
| pKNT25 | A plasmid expressing a target gene on the N-terminal with T25 |  |
| pUT18 | A plasmid expressing a target gene on the N-terminal with T18 |  |
| pUT18C | A plasmid expressing a target gene on the C-terminal with T18 |  |
| pKT25::*graR* | A plasmid expressing GraR on the C-terminal with T25 | This study |
| pKNT25::*graR* | A plasmid expressing GraR on the N-terminal with T25 | This study |
| pUT18::*graR* | A plasmid expressing GraR on the N-terminal with T18 | This study |
| pUT18C::*graR* | A plasmid expressing GraR on the C-terminal with T18 | This study |
| pKNT25::*graS* | A plasmid expressing GraS on the N-terminal with T25 | This study |
| pUT18C::*graS* | A plasmid expressing GraS on the C-terminal with T18 | This study |
| JE2 Δ*graRS* | JE2 devoid of *graR* and *graS* | This study |
| JE2 Δ*graXRS* | JE2 devoid of *graX*, *graR*, *graS* | This study |
| JE2 Δ*graXRS* Δ*stk1* | JE2 Δ*graXRS* with additional deletion of *stk1* | This study |
| JE2 Δ*graX* | JE2 devoid of *graX* | This study |
| JE2 Δ*graX* complement | Chromosomal complementation from JE2 Δ*graX* | This study |
| JE2 Δ*graS* | JE2 devoid of *graS* gene | (2) |
| JE2 Δ*graS* complement | Chromosomal complementation from JE2 Δ*graS* |  |
| pEPSA5 | A plasmid expressing a target gene with xylose-dependent promoter | (6) |
| pEPSA5::*graXRS* | A plasmid expressing *graXRS* with HA-tag and His_6_ tag on C-terminal of GraR | This study |
| pEPSA5::*graXR* | A plasmid expressing *graXR* with HA-tag and His_6_ tag on C-ter. of GraR | This study |
| pEPSA5::*graXRS* with *graS* D35K | pEPSA5::*graXRS* with *graS* D35K mutation | This study |
| pEPSA5::*graXRS* with *graS* r-Hx | pEPSA5::*graXRS* with *graS* r-Hx mutation | This study |
| pEPSA5::*graXRS* with *graS* i-TM | pEPSA5::*graXRS* with *graS* i-TM mutation | This study |
| pEPSA5::*graXRS* with *graS* s-TM | pEPSA5::*graXRS* with *graS* s-TM mutation | This study |
| pEPSA5::*graXRS* with *graR* D51A | pEPSA5::*graXRS* with *graR* D51A mutation | This study |
| pEPSA5::*graXRS* with *graS* H120A | pEPSA5::*graXRS* with *graS* H120A mutation | This study |
| pEPSA5::*graXRS* with *graS* T172A | pEPSA5::*graXRS* with *graS* T172A mutation | This study |
| pEPSA5::*graXRS* with *graS* M119A | pEPSA5::*graXRS* with *graS* M119A mutation | This study |
| pEPSA5::*graXRS* with *graS* H120A/T172A | pEPSA5::*graXRS* with *graS* H120A/T172A mutation | This study |
| pEPSA5::*graXRS* with *graS* H120T/T172H | pEPSA5::*graXRS* with *graS* H120T/T172H mutation | This study |
| pEPSA5::*graXRS* with *graS* H120S | pEPSA5::*graXRS* with *graS* H120S mutation | This study |
| pEPSA5::*graXRS* with *graS* D35K and H120A | pEPSA5::*graXRS* with *graS* D35K and H120A mutation | This study |
| pEPSA5::*graXRS* with *graS* D35K and T172A | pEPSA5::*graXRS* with *graS* D35K and T172A mutation | This study |

1. H. McCarthy, et al., The major autolysin is redundant for Staphylococcus aureus USA300 LAC JE2 virulence in a murine device-related infection model. FEMS Microbiol. Lett. 363 (2016).
2. J. Cho, S. K. Costa, R. M. Wierzbicki, W. F. C. Rigby, A. L. Cheung, The extracellular loop of the membrane permease VraG interacts with GraS to sense cationic antimicrobial peptides in Staphylococcus aureus. *PLoS Pathog.* **17**, e1009338 (2021).
3. M. Arnaud, A. Chastanet, M. Débarbouillé, New Vector for Efficient Allelic Replacement in Naturally Nontransformable, Low-GC-Content, Gram-Positive Bacteria. Appl. Environ. Microbiol. 70, 6887–6891 (2004).
4. M. Falord, G. Karimova, A. Hiron, T. Msadek, GraXSR proteins interact with the VraFG ABC transporter to form a five-component system required for cationic antimicrobial peptide sensing and resistance in Staphylococcus aureus. Antimicrob. Agents Chemother. 56, 1047–1058 (2012).
5. M. G. Olson, M. Goldammer, E. Gauliard, D. Ladant, S. P. Ouellette, A Bacterial Adenylate Cyclase-Based Two-Hybrid System Compatible with Gateway® Cloning. Methods Mol. Biol. Clifton NJ 1794, 75–96 (2018).
6. R. A. Forsyth, *et al.*, A genome-wide strategy for the identification of essential genes in Staphylococcus aureus. *Mol. Microbiol.* **43**, 1387–1400 (2002).

Table S2. Oligonucleotides.

| IDs | Sequences (5’ to 3’, bold: restriction sites) | Ref. |
| --- | --- | --- |
| graS_pMAD Up F | AAA**CCCGGGG**AGTGGAAGTTTAGTGAAAAAATATAC | This study |
| graS_pMAD Up F short | AAA**CCCGGGG**AGTGGAAG |  |
| graS_pMAD Down R | AAA**GGATCC**CTCCTATAATTTATCTTAACTTCATTTCC |  |
| graS_pMAD Down R short | AAA**GGATCC**CTCCTATAATTTATCTTAAC |  |
| graS D35K pMAD Up R | AATAAACTGTCTATTGGAAAATCATACTTGATTAGACTA |  |
| graS D35K pMAD Down F | TAGTCTAATCAAGTATGATTTTCCAATAGACAGTTTATT |  |
| graS D35A pMAD Up R | CTATTGGAAAATCATAAGCGATTAGACTAATGC |  |
| graS D35A pMAD Down F | GCATTAGTCTAATCGCTTATGATTTTCCAATAG |  |
| graS D35E pMAD Up R | CTATTGGAAAATCATAAGCGATTAGACTAATGC |  |
| graS D35E pMAD Down F | GCATTAGTCTAATCGAGTATGATTTTCCAATAGAC |  |
| graS D37K pMAD Up R | AATAAACTGTCTATTGGAAACTTATAATCGATTAGACTA |  |
| graS D37K pMAD Down F | TAGTCTAATCGATTATAAGTTTCCAATAGACAGTTTATT |  |
| graS D41K pMAD Up R | AATAAACTCTTTATTGGAAAATCATAATCGATTAGACTA |  |
| graS D41K pMAD Down F | TAGTCTAATCGATTATGATTTTCCAATAAAGAGTTTATT |  |
| graS F38A pMAD Up R | CAATATAAAATAAACTGTCTATTGG**AGC**ATCATAATCGATTAGACTAATG |  |
| graS F38A pMAD Down F | CATTAGTCTAATCGATTATGAT**GCT**CCAATAGACAGTTTATTTTATATTG |  |
| graS r-Hx pMAD Up R | CAATATCCAAAATATCCAATTATTCATGAGCCATATATCCTTTTC |  |
| graS r-Hx pMAD Down F | GATATATGGCTCATGAATAATTGGATATTTTGGATATTGTTTTTAAACTTC |  |
| graS i-TM pMAD Up R1 | ATTAAGCCAATACTTAGGATGTTCATGCGAGATTTCAAAAAATAAG |  |
| graS i-TM pMAD Up R2 | TCAAAAATAAGTTGAAAAGCATTAAGCCAATACTTAGGATGTTCATG |  |
| graS i-Hx pMAD Down F1 | TTTTGATATGGTTTATATGGGATTATGATTTTCCAATAGACAGTTTATTTTATATTG |  |
| graS i-Hx pMAD Down F2 | GCTTAATGCTTTTCAACTTATTTTTGATATGGTTTATATGGGATTATGAT |  |
| graS r-Hx pMAD Up R1 | TTCAAAGAAACAATATAAAAGTTCATGCGAGATTTCAAAAAATAAG |  |
| graS r-Hx pMAD Up R2 | AAATCATTGTTAAACTTAAATTCAAAGAAACAATATAAAAGTTCATGC |  |
| graS r-Hx pMAD Up R3 | CAAAATATGTCAATAAAAGAAAAATCATTGTTAAACTTAAATTCAAAGAAAC |  |
| graS r-Hx pMAD Up R4 | GTCTATTGGAAAATCATAATCAAAATATGTCAATAAAAGAAAAATCATTGTTA |  |
| graS r-Hx pMAD Up R5 | CCAAAATATCCATAAACTGTCTATTGGAAAATCATAATCAAAATATGTC |  |
| graS r-Hx pMAD Down F1 | GTTAGGCATTAGTCTAATCAAAGAAGTAAAATTATATAAGCATTTTGACAA |  |
| graS r-Hx pMAD Down F2 | ATTGTTTTTAAACTTCCTTATGTTAGGCATTAGTCTAATCAAAGAAG |  |
| graS r-Hx pMAD Down F3 | CAGTTTATGGATATTTTGGATATTGTTTTTAAACTTCCTTATGTTAGG |  |
| graS r-Hx pMAD Down F4 | GATTATGATTTTCCAATAGACAGTTTATGGATATTTTGGATATTGTTTTTAAAC |  |
| graS H pMAD Up F | AAACCCGGGGAAATATACTAAATGATATTGGGTGATATGGATGC |  |
| graS H pMAD Up F short | AAACCCGGGGAAATATACTAAATG |  |
| graS H pMAD Down R | AAAGGATCCGACCCATAATAGCAATAAACTCGCCTTCTTC |  |
| graS H pMAD Down R short | AAAGGATCCGACCCATAATAG |  |
| graS H120A pMAD Up R | CTGTAATGGTTTGTTCTGCCATGTTCAATTGCAAC |  |
| graS H120A pMAD Down F | GTTGCAATTGAACATGGCAGAACAAACCATTACAG |  |
| graS T172A pMAD Up R | GAGATTCTAATCTAGCAATATACAGCTGTG |  |
| graS T172A pMAD Down F | CACAGCTGTATATTGCTAGATTAGAATCTC |  |
| graS M119A pMAD Up R | GTAATGGTTTGTTCATGTGCGTTCAATTGCAACTG |  |
| graS M119A pMAD Down F | CAGTTGCAATTGAACGCACATGAACAAACCATTAC |  |
| graS H120T pMAD Up R | CTGTAATGGTTTGTTCAGTCATGTTCAATTGCAAC |  |
| graS H120T pMAD Down F | GTTGCAATTGAACATGACTGAACAAACCATTACAG |  |
| graS T172H pMAD Up R | CGTTGAGATTCTAATCTATGAATATACAGCTGTGTATC |  |
| graS T172H pMAD Down F | GATACACAGCTGTATATTCATAGATTAGAATCTCAACG |  |
| graS H120S pMAD Up R | CTGTAATGGTTTGTTCAGACATGTTCAATTGCAAC |  |
| graS H120S pMAD Down F | GTTGCAATTGAACATGTCTGAACAAACCATTACAG |  |
| graS_F for pKNT25, pKT25, pUT18, and pUT18C | AAAGGATCCCAATAATTTGAAATGGGTAGCTTATTTTTTG | This study |
| graS R for pKNT25 and pUT18 | AAAGAATTCGAAAATGACAAATTTGTCACTTCCG |  |
| graS R for pKT25 and pUT18C | AAAGAATTCTTAAAATGACAAATTTGTCACTTCC |  |
| graR F for pKNT25, pKT25, pUT18, and pUT18C | AAAAGGATCCCCAAATACTACTAGTAGAAGATGACAATAC |  |
| graR R for pKNT25 and pUT18 | AAAAGAATTCGATTCATGAGCCATATATCCTTTTC |  |
| graR R for pKT25 and pUT18C | AAAAGAATTCTTATTCATGAGCCATATATCCTTTTC |  |
| pMAD Up F for Δ*graRS* | AAA**CCCGGG**GTAACTAAAAGGTGGAGTAATATG |  |
| pMAD Up R for Δ*graRS* | CCGACATGCGTTCGTCATCTTCTACTAGTAGTATTTGC |  |
| pMAD Down F for Δ*graRS* | GTAGAAGATGACGAACGCATGTCGGAAGTGAC |  |
| pMAD Down R for Δ*graRS* | AAA**GGATCC**GCGTATTTTAATGCTACAAAGCTAAAATAC |  |
| pMAD Up F for Δ*graRS* short | AAA**CCCGGG**GTAACTAAAAG |  |
| pMAD Down R for Δ*graRS* short | AAA**GGATCC**GCGTATTTTAATG |  |
| pMAD Up F for Δ*graXRS* | AAA**CCCGGG**CGTACAAGATTATGGAATTAAGCG |  |
| pMAD Up R for Δ*graXRS* | TAAAATGACAAATTTGTCACACCTGCTAATAAAACTTTAGGTTTC |  |
| pMAD Down F for Δ*graXRS* | CTAAAGTTTTATTAGCAGGTGTGACAAATTTGTCATTTTAAACATG |  |
| pMAD Down R for Δ*graXRS* | AAA**GGATCC**GGCATAATGTGATAAATTTTGACG |  |
| pMAD Up F for Δ*graX* | AAA**GGATCC**TATGGAATTAAGCGTAAAGGTAGG |  |
| pMAD Up R for Δ*graX* | AAA**CCCGGG**AATATATCCTGTTCCACCTGCTA |  |
| pMAD Down F for Δ*graX* | AAA**CCCGGG**TACTTTATACAAGCACCAATGCA |  |
| pMAD Down R for Δ*graX* | AAA**GGATCC**GAAATGGCGTTTCCGCTAAAT |  |
| pMAD Up F for Δ*stk1* | AAA**CCCGGG**GTTACTTTTGAATATGGTCTGTTTG |  |
| pMAD Up R for Δ*stk1* | GCTACAGTTTTACCCTTATCTACAATTTTATATCGTTCATTTATTATTTTAC |  |
| pMAD Down F for Δ*stk1* | GATATAAAATTGTAGATAAGGGTAAAACTGTAGCTGAAAAAGAAG |  |
| pMAD Down R for Δ*stk1* | AAA**GGATCC**CTAAACTTACATGTTTCACCATATCG |  |
| stk1 IDF F | GATGAAGATATTCAAATTAATTTTGCTG |  |
| stk1 IDF R | CCCTATCTCTAATTGATGCTTAAC |  |
| stk1_IDF1 | GTGATCACTCATTTGTTAATCATC |  |
| graXRS & XR for pEPSA5 F1 | AAAAAA**GAGCTC**TGGTTACAGTTTAAAGGAGTATTTTAG |  |
| graXRS for pEPSA5 F2 | TGGTTACAGTTTAAAGGAGTATTTTAG |  |
| graXRS for pEPSA5 R1 | CTGGAACATCATATGGATATTCATGAGCCATATATCCTTTTCC |  |
| graXRS for pEPSA5 R2 | CACCCAATATTAAGCATAATCTGGAACATCATATGGATATTCATG |  |
| graXRS for pEPSA5 F3 | CATATGATGTTCCAGATTATGCTTAATATTGGGTGATATGGATGAAT |  |
| graXRS for pEPSA5 F4 | CTTAATATTGGGTGATATGGATGAATAATTTGAAATGGGTAGCT |  |
| graXRS for pEPSA5 R3 | TTAAAATGACAAATTTGTCACTTCC |  |
| graXRS for pEPSA5 R4 | AAAAAA**TCTAGA**TTAAAATGACAAATTTGTCACTTCC |  |
| graXR for pEPSA5 R | AAA**TCTAGA**TTAAGCATAATCTGGAACATCATATG |  |
| IDF for pEPSA5 F | TAGATATCTCGGACCGTCATAAAAAATT |  |
| IDF for pEPSA5 R | CAAATTCTGTTTTATCAGACCGCT |  |
| IDF 1 for graXRS mutation | GTTCATCACTTTCAGCAACGA | (1) |
| IDF 2 for graXRS mutation | TAGATCAACGATGAAGGTAGTTTT |  |
| IDF 3 for graXRS mutation | AAATTACAAGCGATTTATCGTCGT |  |
| IDF 4 for graXRS mutation | TCAAGAAAGAAAACAAGCATTACTA |  |
| IDF 5 for graXRS mutation | AATATATGGAACTAAAAAAATGGCACA |  |
| IDF 6 for graXRS mutation | TACGAGTGTAGTATTGTATTTTAGCTTTG |  |
| IDF 7 for graXRS mutation | GTAAATGATGTCATGTTCTCATCA |  |
| IDF 8 for graXRS mutation | CTGTATGTTGCATTATATACATAAAGC |  |
| mprF for specific primer | TTATTTGTGACGTATTACAC | This study |
| mprF for qRT-PCR F | TCACAGTGGCGACATTCTTC |  |
| mprF for qRT-PCR R | TCTTACTGGGCGTTTCAACC |  |
| gyrB for specific primer | TTAGAAGTCTAAGTTTGCAT |  |
| gyrB for qRT-PCR F | GGTGCTGGGCAAATACAAGT |  |
| gyrB for qRT-PCR R | TCCCACACTAAATGGTGCAA |  |
| pMAD IDF F | GTTACGTTACACATTAACTAGACAG |  |
| pMAD IDF R | GAAGAATCATAATGGGGAAGG |  |
| graX IDF F | AAATACTATGCATTGGCAATGGAAC |  |
| graX IDF R | TAATTTCATAGCTGTCACAGGTGTT |  |
| graRS IDF F | GTTTAAAATGACAAATTTGTCACTTCCG |  |
| graRS IDF R | GACTTGTGAGCCTTCCTTTA |  |
| IDF F for pKT25 | GATATCGACATGTTCGCCA |  |
| IDF F for pUT18C | CTATGTCTTCTACGAGAACCG |  |
| IDF F for pUT18 and pKNT25 | GTTTCCCGACTGGAAAGC |  |
| IDF R for pKT25 | GGCGATTAAGTTGGGTAAC |  |
| IDF R for pUT18 | GACATTAACCTATAAAAATAGGCGTATC |  |
| IDF R for pKNT25 | CCGAACAGTTTGGAAAGATTC |  |
| IDF R for pUT18C | CTATAAAAATAGGCGTATCACGAG |  |

1. J. Cho, S. K. Costa, R. M. Wierzbicki, W. F. C. Rigby, A. L. Cheung, The extracellular loop of the membrane permease VraG interacts with GraS to sense cationic antimicrobial peptides in Staphylococcus aureus. PLoS Pathog. 17, e1009338 (2021).
